# Reticulate evolution as a management challenge: Patterns of admixture with phylogenetic distance in endemic fishes of western North America

**DOI:** 10.1111/eva.13042

**Published:** 2020-06-29

**Authors:** Max R. Bangs, Marlis R. Douglas, Patrick C. Brunner, Michael E. Douglas

**Affiliations:** ^1^ Department of Biological Sciences University of Arkansas Fayetteville AR USA; ^2^ Department of Biological Sciences Florida State University Tallahassee FL USA; ^3^ Integrative Biology Swiss Federal Institute of Technology (ETH) Zürich Switzerland

**Keywords:** adaptive management, ddRAD, hybridization, introduced species, introgression, reproductive isolation, species of concern

## Abstract

Admixture in natural populations is a long‐standing management challenge, with population genomic approaches offering means for adjudication. We now more clearly understand the permeability of species boundaries and the potential of admixture for promoting adaptive evolution. These issues particularly resonate in western North America, where tectonism and aridity have fragmented and reshuffled rivers over millennia, in turn promoting reticulation among endemic fishes, a situation compounded by anthropogenic habitat modifications and non‐native introductions. The melding of historic and contemporary admixture has both confused and stymied management. We underscore this situation with a case study that quantifies basin‐wide admixture among a group of native and introduced fishes by employing double‐digest restriction site‐associated DNA (ddRAD) sequencing. Our approach: (a) quantifies the admixed history of 343 suckers (10 species of Catostomidae) across the Colorado River Basin; (b) gauges admixture within the context of phylogenetic distance and “ecological specialization”; and (c) extrapolates potential drivers of introgression across hybrid crosses that involve endemic as well as invasive species. Our study extends across an entire freshwater basin and expands previous studies more limited in scope both geographically and taxonomically. Our results detected admixture involving all 10 species, with habitat alterations not only accelerating the breakdown of reproductive isolation, but also promoting introgression. Hybridization occurred across the genus despite phylogenetic distance, whereas introgression was only detected within subgenera, implicating phylogenetic distance and/or ecological specialization as drivers of reproductive isolation. Understanding the extent of admixture and reproductive isolation across multiple species serves to disentangle their reticulate evolutionary histories and provides a broadscale perspective for basin‐wide conservation and management.

## 1 INTRODUCTION

Reticulated evolution is a product of several, often interacting phenomena, including horizontal gene transfer, polyploidization, and hybridization with introgression (Wendel & Doyle, 1998). All have been traditionally viewed as examples of “aberrant evolution,” in that their occurrence was disruptive to the process of adaptation and speciation, with results translated as a network rather than a more traditional bifurcating tree. This supposition of aberrancy is best reflected in more legacy perspectives ([i.e., “… the grossest blunder in sexual preference which we can conceive of an animal making”; Fisher, 1930:130] and [“… the infection of one species with the genes from a second”; Du Rietz, 1930:376, 380, 386, 411]).

Rather than an evolutionary contradiction, hybridization, defined as the mixing of two species, offers instead an opportunity to grasp how evolution has been facilitated, in lieu of reproductive isolation (Good, Demboski, Nagorsen, & Sullivan, 2003). Hybridization, especially when coupled with introgression (i.e., the incorporation of alleles from one species into the gene pool of another), has long been thought to play a beneficial evolutionary role in both plants (Arnold, 1992) and animals (Dowling & Secor, 1997). It can promote evolution by (a) generating new genetic variation, (b) transferring adaptive traits, and (c) producing new lineages that exploit a novel niche within which neither parental taxa could succeed (Darras, Leniaud, & Aron, 2014; Edelman et al., 2019; Seehausen et al., 2014).

At the same time, it can have negative consequences, as with anthropogenic introductions, by either disrupting local adaptations or genetically swamping endemics, leading to the effective extinction of a species (Rhymer & Simberloff, 1996). These conflicting views have often complicated conservation and management (Allendorf, Leary, Spruell, & Wenburg, 2001), to include policies on how to adjudicate (Haig & Allendorf, 2006; vonHoldt, Brzeski, Wilcove, & Rutledge, 2018).

Over the last 20+ years, genetic data have helped to inform biodiversity management, with both methodological and analytical approaches becoming more sophisticated. Genomics has been repeatedly advocated as a mechanism to better understand the complexities of conservation issues (Funk, McKay, Hohenlohe, & Allendorf, 2012), yet easy solutions are not apparent. For example, the appropriate application of genomic tools has become somewhat contentious (Benestan et al., 2016), with a common thread being the necessity for a practical, management‐oriented approach (Garner et al., 2016).

In this regard, one issue of historic importance that would benefit from increased resolution is the occurrence and extent of admixture in natural populations (Allendorf et al., 2001). This practical problem fits easily into an evolutionary framework, particularly in relation to (a) quantifying the genetic erosion induced by invasive species (Lowe, Mulfeld, & Allendorf, 2015; Rhymer & Simberloff, 1996); (b) identifying cryptic species (Devitt, Wright, Cannatella, & Hillis, 2019); and (c) parsing admixture among endemics that stems from anthropogenic impacts (Abbott, Barton, & Good, 2016; Hamilton & Miller, 2016).

Over the past 20 years (Box 1), one focus of our team has been centered on desert fishes of the American Southwest, most recently by applying genomic methods to provide insights on species of conservation concern (Box 2). The arid southwest has been one of the most impacted environments, with demands for water driving both policy and socioeconomic agendas further exacerbated by climate‐driven drought (Ficke, Myrick, & Hansen, 2007; Hinck, 2007). Here, we use the opportunity to illustrate the conservation challenges surrounding catostomids and how genomic tools can help clarify the manner by which hybridization and introgression have impacted three endemic species that face the combined threat of habitat alterations and introduced species.

Finescale Suckers (genus *Catostomus*) are known to hybridize, especially when invasive congeners have been introduced and/or habitats modified (Holden & Stalnaker, 1975, Douglas & Douglas, 2010). Introgression is also known to have occurred throughout the history of the genus as suggested by discordance between mitochondrial and morphological data (Smith, Stewart, & Carpenter, 2013; Unmack et al., 2014) and confirmed by genomic data (Bangs, Douglas, Mussmann, & Douglas, 2018), indicating a history of natural hybridization and introgression. Taken together, hybridization can occur between both endemics and introduced species and may have more far‐reaching, albeit subtle effects such as potentially providing a bridge for introgression among native species that would not naturally hybridize (McDonald, Parchman, Bower, Hubert, & Rahel, 2008), although the extent of this “hybrid bridge” has come under recent question (Mandeville, Parchman, McDonald, & Buerkle, 2015; Mandeville et al., 2017). These studies of hybridization, however, tend to focus on regional scales and with focus on two or three species at a time. Here, we expand on this work to examine an entire basin to include all species that are endemic or introduce in order to string together past work and elucidate larger patterns of hybridization and introgression.

**Figure 1:**
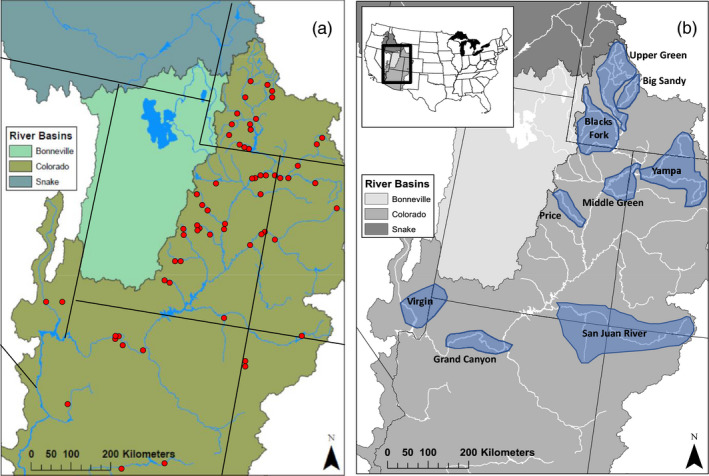
(a) Map of the Colorado River and adjacent basins. Those found in this study are highlighted in blue; (b) sample sites depicted with regard to drainage and basin. FGD, Flaming Gorge Dam; GCD, Glen Canyon Dam

The study area extends across the entire Upper Colorado River Basin (Figure 1a) and encompasses four native species: Flannelmouth Sucker (FMS; *Catostomus latipinnis*), Bluehead Sucker (BHS; *C. Pantosteus discobolus*), Mountain Sucker (MTS; *C. P. platyrhynchus*), and Razorback Sucker (RBS; *Xyrauchen texanus*) (Figure 1b). All exhibit complex patterns of admixture that reflect both contemporary, as well as historic introgression facilitated by geological changes (Box 3). We describe the extent of this hybridization and introgression among these four endemic species as well as with two species native to the Lower Colorado River Basin [Sonora Sucker (SOS; *C. insignis*) and Desert Sucker (DES; *C. P. clarkii)*] and with two non‐native species [White Sucker (WTS; *C. commersonii*) and Longnose Sucker (LNS; *C. catostomus*)].

Our study is based upon 20+ years of sampling and represents the first range‐wide molecular evaluation of hybridization and introgression involving all members of a clade that occur in the basin, both native and introduced. As such, it provides a blueprint for management how to disentangle contemporary events from those historic. Our results identify the breadth of invasive‐endemic hybridizations and clarify the manner by which it is facilitated via habitat fragmentation, a second anthropogenic impact. We also bookmark the historic legacy of admixture among native species. Finally, we employ our results to define reproductive isolation among our study species as a component of phylogenetic distance, or potentially, as a stepping stone to ecological speciation.

Box 1 **The shaping of professional trajectories**

Two of us (MRD and PCB) were in the inaugural cohort of graduate students Louis mentored as a young, but visionary assistant professor at Laval University, in Quebec. Sadly, Patrick is no longer with us to make his own voice heard. I reflect on the opportunities Louis provided to both of us and how they positively affected our careers. I offer these thoughts as impetus for current graduate students and postdoctoral fellows to pursue their aspirations, but also to remind established professionals of their opportunity to promote younger colleagues. Thinking about my graduate years working with Louis made me to realize in retrospect how influential he was in shaping my professional growth as a scientist. I detail these in five vignettes below:

1. Facilitate and Provide Opportunities
  Patrick and I were two Swiss graduate students with ideas and aspirations, but without the skills or environment to achieve them. Although we received funding from the Swiss government to conduct conservation genetics studies on salmonids in Switzerland, we could not have completed them without the generous opportunity offered by Louis.
  My PhD research on *Coregonus*, and Patrick's on *Salvelinus*, occurred in the Central Alpine lakes at the dawn of the microsatellite DNA era. We were fortunate to meet Louis Bernatchez, then a newly minted faculty at Laval, who kindly opened his laboratory to us. This was a tremendous opportunity, in that no one in Switzerland at that time employed this methodology. *Coregonus* in Swiss lakes has become a prime model for fish “species flocks,” and this would not have occurred if Louis had not shared his knowledge of molecular techniques with two Swiss students and instilled in them his enthusiasm for biodiversity conservation.
2. Make it Work.
  Louis’ 1st laboratory was a single room (~20 m^2^) where 10 of us literally worked elbow‐to‐elbow doing DNA extractions, allozymes, RFLP electrophoresis, and sequencing. A single bench in another laboratory was dedicated to PCR setup. Note: This was the early '90s—PCR was just being adopted as a standard method in fisheries genetics and automated sequencers were not yet available. Although it was a bit crowded at times, the group made it work with a shared camaraderie and purpose in generating solid science. This is a tribute to what enthusiasm and an entrepreneurial spirit can achieve.
3. Do it Right
  Even though the early laboratory was small, it worked because Louis established an efficient workflow, subsequently adopted in my own laboratories: Workspace was assigned to tasks that employed standardized protocols. Metadata were recorded on standardized forms, rather than individual notebooks, to ensure consistency across long‐term projects.
  Louis provided guidance when needed and was an invested, but hands‐off advisor. He achieved productivity by making resources available and challenging us to give our best. We prospered in such an environment; it requires independence and self‐motivation and is successful when mentees set high standards for themselves. I adopted this approach in my own mentoring, but also realized that such a “free spirited” environment works for some but not all.
4. Think Big—and Outside the Box
  Louis always thinks “big” and did not let convention limit the goals he set for his team. Over his career, Louis not only had a huge impact on Conservation Genetics, but also helped shape the emerging field of Molecular Ecology. In this sense, we not only received a great start by being his students, but also prospered beyond our graduate years by tracking the slipstream of his ideas and innovations. Louis inspired us to think outside the box and not be confined by circumstance or dogma. We were pushed to “think outside the box” and pursue novel ideas, but also to generate solid data and always consider alternative hypotheses when interpreting results.
5. To Go Boldly
  Embracing new opportunities is a key aspect of my professional trajectory. Each change demonstrated that taking reasonable professional chances will benefit in the long run. The hard part for me was to convince myself to take that next big step. In this sense, I moved among major institutions (two each for PhD and postdocs and three for faculty positions). I am currently an endowed professor at the University of Arkansas. For sure, each transition was challenging, but each provided amazing opportunities. This all began in the laboratory of Louis Bernatchez. His welcoming and entrepreneurial spirit guided me professionally. From feedback by my own former students now in established careers, I also realize that it is not the “big things” that guide them throughout their careers, but rather those small actions that resonate most with young students and inspire them to go boldly.

Box 2 **Admixture in Southwestern Fishes**

One of the major challenges in conservation today is how to deal with the complexities of hybridization and reticulate evolution (Allendorf, Hohenlohe, & Luikart, 2010). Historically, hybridization has been viewed as a negative, and thus, management has focused on removal of hybrids. However, recent genomic work has highlighted the importance of reticulate evolution in the emergence of biological diversity and adaptation of species leading to management perspectives that accept it as a key process (Hamilton & Miller, 2016).

Our team has been active in this regard. For example, we recently leveraged genomic techniques (i.e., ddRAD) as a means of quantifying reticulate evolution in a complex of large‐bodied minnows (*Gila*) found in the Upper Colorado River Basin of Western North America (Chafin, Douglas, Martin, & Douglas, 2019). We also applied these techniques to (a) define species boundaries in a similar but more taxonomically diverse complex in the Lower Colorado River Basin (Chafin, Douglas, Bangs, Mussmann, & Douglas, 2019); (b) disentangle the phylogeny of *Catostomus* by testing hypotheses that were established to its evolutionary trajectory (Bangs et al., 2018), and (c) delineate species of conservation concern (Bangs, Douglas, Chafin, & Douglas, 2020). These studies, and others, actively promote ongoing management by defining historic introgression and contemporary hybridization in native fishes, as well as developing a genetic database that can be used to accurately parse contemporary introgression among species.

Suckers (family Catostomidae) readily hybridize, as do many cypriniform fishes, and particularly the genus *Catostomus*, a situation exacerbated anthropogenically by introducing invasive congeners and the extensively modifying riverine habitat (Bangs, Douglas, Thompson, & Douglas, 2017; Douglas & Douglas, 2010; Holden & Stalnaker, 1975). This phenomenon has also been hypothesized to include more subtle effects, such as providing a “hybrid bridge” for introgression among species that would not do so naturally (McDonald et al., 2008). Hybridization without the influence of introduced congeners has been observed in native sympatric fishes (Hubbs, Hubbs, & Johnson, 1943; Nelson, 1968) and seemingly occurs between genera within families (Buth, Haglund, & Minckley, 1992; Dowling et al., 2016; McAda & Wydoski, 1980; Tranah & May, 2006). However, the manner by which these genera should be taxonomically categorized is the subject of debate (Bangs et al., 2018).

Box 3 **The ecological theater of Western North America**

The geomorphic history of western North America (synopsized from Minckley, Hendrickson, & Bond, 1986; Spencer, Smith, & Dowling, 2008) has catalyzed the evolution of its resident aquatic fauna. The tectonics of the region have alternately fractured and coalesced drainages, consequently reshuffling the distributions of aquatic species over time. For example, the Basin and Range physiographic province spanned much of Western North America during Miocene (Figure 1a) and was replete with small‐bodied species that were subsequently coalesced by vicariant tectonism into reproductively isolated refugia. Antecedent streams (i.e., those previously formed) on the adjacent Colorado Plateau (Spencer et al., 2008, Figure 1) deeply incised the Plateau as it uplifted, eroding headwater canyons and subsequently isolating not only aquatic but terrestrial fauna as well (Douglas et al., 2016). During this process, other streams drained internally to form several closed Plateau lakes that eventually emptied into the newly formed Colorado River as it transitioned across the Basin and Range, circa 5 mya. This allowed a rather depauperate assemblage of lacustrine‐evolving, larger‐bodied species (Uyeno & Miller, 1965) to disperse downstream into diverse habitats replete with new ecological niches.

Quaternary glaciation in western North America was limited to high elevation peaks, in contrast to its overwhelming presence in Eastern North America as the Laurentide Ice Sheet. Nevertheless, climate processes still resonated in the west, with broad impacts on resident biodiversity (Douglas, Douglas, Schuett, & Porras, 2006). The western North American monsoon was severely depressed by glaciation (Bhattacharya, Tierney, Addison, & Murray, 2018), such that summer temperatures in the continental interior averaged 8–14°C below contemporary levels, while plant communities were depressed 600–1,200 m in elevation. Pinyon Pine and Juniper were largely predominated on the landscape, slowly transitioning over time to open woodlands as climate changed. Desert vegetation was restricted to <300 m elevation (Death Valley, California, and confluence of the Sea of Cortéz), but soon expanded in tandem with an extensive Holocene drought (Sullivan et al., 2016). These climatic effects were global in nature and greatly impacted aquatic and terrestrial fauna beyond Western North America (Brunner, Douglas, & Bernatchez, 1998; Brunner, Douglas, Osinov, Wilson, & Bernatchez, 2001; Bryson, Murphy, Lathrop, & Lazcano‐Villareal, 2011; Migliore et al., 2019; Morales‐Barbero, Martinez, Ferrer‐Castán, & Olalla‐Tárraga, 2018).

In contrast to vicariant events, the Quaternary also provoked frequent admixture as glacial periods were supplanted by warmer interglacials (nine of which were recorded during the last 0.8 mya; Douglas, Douglas, Schuett, & Porras, 2009). Drainage reorganization (i.e., stream captures, diversions, beheadings) occurred frequently during these more pluvial periods, and not only extended fish distributions into adjacent but previously isolated basins, but also promoted admixture that subsequently confounded taxonomy (Dowling et al., 2016; Smith, Hall, Koehn, & Innes, 1983). Of late, this situation has been exacerbated in Western North America by ongoing admixture between endemic and introduced fishes, with management alternatives limited due to the weak resolution provided by legacy approaches. Again, interglacials were global in their occurrence and served to promote gene flow and secondary contact on a broadscale (Douglas & Brunner, 2002; Douglas, Brunner, & Bernachez, 1999; Ericson et al., 2019; Kohli, Fedorov, Waltari, & Cook, 2015; Licona‐Vera, Ornelas, Wethington, & Bryan, 2018).

## 2 METHODS

### 2.1 Sample acquisition

This range‐wide study was made possible by collaborative sampling efforts conducted over 25+ years. Here, we attempt to disentangle historic versus contemporary signals of reticulate evolution in suckers of the Colorado River Basin. We employed reduced genomic approaches to analyze DNA extracted from fin clips and tissue plugs gathered across the basin during 1995–2015 (Douglas, Brunner, & Douglas, 2003; Douglas & Douglas, 2010; Douglas & Marsh, 1998; Hopken, Douglas, & Douglas, 2013). Additional samples were obtained from the Museum of Southwestern Biology (University of New Mexico). A total of 409 samples were used and included 343 samples captured in areas of known hybridization and 66 samples from outside of these areas that are known to be pure based on previous phylogenomic and population genetic work (Bangs et al., 2018, 2020) and served as reference to verify species identification. The 343 samples that encompassed potential hybridization included both field‐identified pure samples and field‐identified hybrids and were collected across nine regions (Figure 1b): (a) Big Sandy River (Wyoming: *N* = 45), (b) Blacks Fork (Wyoming: *N* = 50), (c) Upper Green River (Wyoming: *N* = 27), (d) Middle Green River (Utah: *N* = 11), (e) Yampa River (Colorado/Utah: *N* = 60), (f) Price River (Utah: *N* = 25), (g) San Juan River (New Mexico/Utah: *N* = 47), (h) Grand Canyon (Arizona: *N* = 67), and (i) Virgin River (Utah/Nevada: *N* = 11). Details with regard to species, samples, field identification, and regions are in Appendix A and are shown in Figure 1.

To properly assess hybridization, it is important to have a good reference database for the parental species. This study was made possible by our previous phylogenomic work (Bangs et al., 2018) that provided us with a solid reference database for parental species outside of the known hybridization areas. In addition to field‐identified hybrids (*N* = 115), we included field‐identified pure parental species (*N* = 228). This allowed us to test for genetic structure among natural populations that could be indicative of cryptic variation in comparison with our phylogenomic reference database. It also made it possible to verify potential cryptic hybridization that might not have been captured by simple field identifications.

### 2.2 Data collection

Genomic DNA was extracted with PureGene^®^ Purification Kit or DNeasy^®^ Tissue Kit (Qiagen Inc., Valencia CA) and stored in DNA hydrating solution (same kits). Libraries for double‐digest restriction site‐associated DNA (ddRAD) were generated following the protocol outlined in Bangs et al. (2018). This included digesting with *Pst*I (5′‐CTGCAG‐3′) and *Msp*I (5′‐CCGG‐3′), pooling 48 barcoded individuals prior to a size selection of 350–400 bps, PCR amplification, and combining two libraries per lane of Illumina HiSeq 2000 single‐end 100‐bp sequencing. Samples for each reference species, region, and hybrid type were randomly distributed across several libraries and lanes so as to reduce the potential bias in library preparation or lane effects. Sequencing was performed at the University of Wisconsin Biotechnology Center (Madison).

### 2.3 Filtering and alignment

Illumina reads were filtered and aligned using pyrad v.3.0.5 (Eaton & Ree, 2013) following the parameters determined in our previous ddRAD work in this system (Bangs et al., 2018). This included: removal of restriction site sequences and barcodes, and clustering at a threshold of 80% based the uncorrected sequence variation in catostomid fishes (Bangs et al., 2017; Chen & Mayden, 2012). In addition, loci were removed if they displayed: (a) <5 reads per individual, (b) >10 heterozygous sites within a consensus, (c) >2 haplotypes for an individual, (d) >75% heterozygosity for a site among individuals, and (e) <50% of individuals at a given locus. Filter 1 reduces the chance for false homozygosity, filters 2–4 remove paralogs, and filter 5 decreases the amount of missing data. This filtering process has worked well in our previous work (Bangs et al., 2018, 2020) for phylogenetic, population genetic, and hybrid analyses.

### 2.4 Clustering algorithms

All analyses employed unlinked SNPs generated by pyrad, which samples one SNP at random from each RAD locus. Bayesian clustering (structure v. 2.3.4; Pritchard, Stephens, & Donnelly, 2000) employed the admixture model with correlated allele frequencies and a burn‐in of 100,000 generation, followed by 500,000 generations post‐burn‐in. No population priors were employed. Genetic clusters (k = 1–16) were each run with 15 iterations and then averaged across iterations to determine final values. We resolved the most likely genetic cluster by using the estimated log probability of data Pr(*x*|*k*) and the Δ*k* statistic (per Evanno, Regnaut, & Goudet, 2005).

Average pairwise genetic distances were calculated between all species using the complete sequence alignment for all 14,007 loci (Bangs et al., 2018). Distances were calculated using the default F84 model in dnadist, as implemented in phylip (Felsenstein, 1993).

### 2.5 Hybrid detection

For hybrid analyses, we used unlinked SNPs with additional filtering to include only fixed differences between the two parental species and the removal of loci that contained <80% individuals. Confirmation of pure parental species for determining fixed SNPs was based on *q* = 1.0 for a single cluster (species) in the Bayesian clustering analysis above. Only fixed differences between species were used to ensure accurate interspecific heterozygosity. Both hybrid analyses require the designation of parental populations, with only two parental species per test.

We developed a hybrid index (Gompert & Buerkle, 2009) for each cross by implementing the R‐package introgress (Gompert & Buerkle, 2010). This involved a test of hybridization between the following species: (a) Flannelmouth × White (FMS × WTS), (b) Bluehead × White (BHS × WTS), (c) Flannelmouth × Bluehead (FMS × BHS), (d) Bluehead × Longnose (BHS × LNS), (e) White × Longnose (WTS × LNS), (f) Bluehead × Mountain (BHS × TS), (g) Bluehead × Desert (BHS × DES), (h) Bluehead × Razorback (BHS × RBS), (i) Flannelmouth × Razorback (FMS × RBS), and (j) Flannelmouth × Sonora (FMS × SOS). The same package (above) was used to create a triangle plot depicting hybrid index by interspecific heterozygosity for each admixture test and (occasionally) by location as well.

We then utilized newhybrids (Anderson & Thompson, 2002) to test the probability of assignment to a hybrid class, including first‐filial (F1), second‐filial (F2), and first‐ and second‐generation backcross (Bx). Additional crossings, while of interest, would fail to assign individuals to any of the designed hybrid or parental categories. Only first‐ and second‐generation backcrosses were so designated, given the potential for ancestral crosses to be spuriously assigned to later‐generation backcross categories (i.e., third and fourth). If this occurred, individuals would then be erroneously designated as more contemporaneous.

## 3 RESULTS

Some 11,669 loci were obtained postfiltering. These contained 89,868 SNPs of which 66,151 were parsimoniously informative, with 32.39% missing data. Average coverage was 19×, with all individuals > 11.5× coverage and <80% missing data. We utilized 11,501 unlinked SNPs in our Bayesian clustering runs. The total number of fixed SNPs, number of individuals, and amount of missing data for each hybrid cross are presented in Table 1.

**Table 1:** Number of fixed SNPs used for hybrid analysis between species pairs (Cross) of *Catostomus* (Pisces: Catostomidae)

Cross	SNPs	% Missing	# indiv	# hybrid
FMS × WHS	260	13.3	108	68
FMS × SOS	403	11	51	1
FMS × RBS	399	10.1	44	6
BHS × FMS	302	12.6	58	7
BHS × WHS	253	14.1	73	29
BHS × LNS	251	12.7	34	2
BHS × RBS	232	10.4	7	1
BHS × DES	99	10	100	2
BHS × MTS	274	11.6	144	17
WHS × LNS	477	9.5	8	1

Note

These include all samples that clustered to only the two species listed in the cross, whether pure or hybrid. Abbreviations for crosses are BHS, Bluehead Sucker, DES, Desert Sucker, FMS, Flannelmouth Sucker, LNS, Longnose Sucker, MTS, Mountain Sucker, RBS, Razorback Sucker, RGS, Rio Grande Sucker, SOS, Sonora Sucker, UTS, Utah Sucker, WHS, White Sucker. Also included are percent missing data (% Missing), number of individuals (# indiv), and number of samples identified as admixed by structure (# hybrid).

### 3.1 Bayesian clustering

The most likely number of genetic clusters (gene pools) was *k* = 10, corresponding to the 10 species in our study (Figure S1). All 66 reference samples from outside the known hybridization area were assigned to a single cluster (Figure S2). All 115 field identify hybrids had mixed assignments as did 19 field‐identified pure specimens. These included (a) eight Bluehead Sucker × Mountain sucker hybrids in the Price River; (b) one from the Virgin River that displayed Desert Sucker × Bluehead Sucker ancestry; (c) one Flannelmouth Sucker from the Virgin River assigned to the Flannelmouth Sucker cluster, but reflected introgression with Sonora Sucker); (d) six hybrids in the Grand Canyon that included one Bluehead Sucker × Desert Sucker hybrid, one Flannelmouth Sucker × Sonora Sucker hybrid, and four Flannelmouth Sucker × Razorback Sucker hybrids; and (e) three Flannelmouth × Sonora Sucker hybrids in the Upper Green River. Most of these samples (*N* = 16) were collected without hybridization in mind (Grand Canyon, Virgin River, and Price River). The other three samples from the Upper Green River were identified as Flannelmouth Sucker but instead had low amount of introgression (*q* < 0.05) with White Sucker. These were subsequently excluded as pure reference samples in our hybrid analyses and allocated to the hybrid category instead.

**Figure 2:**
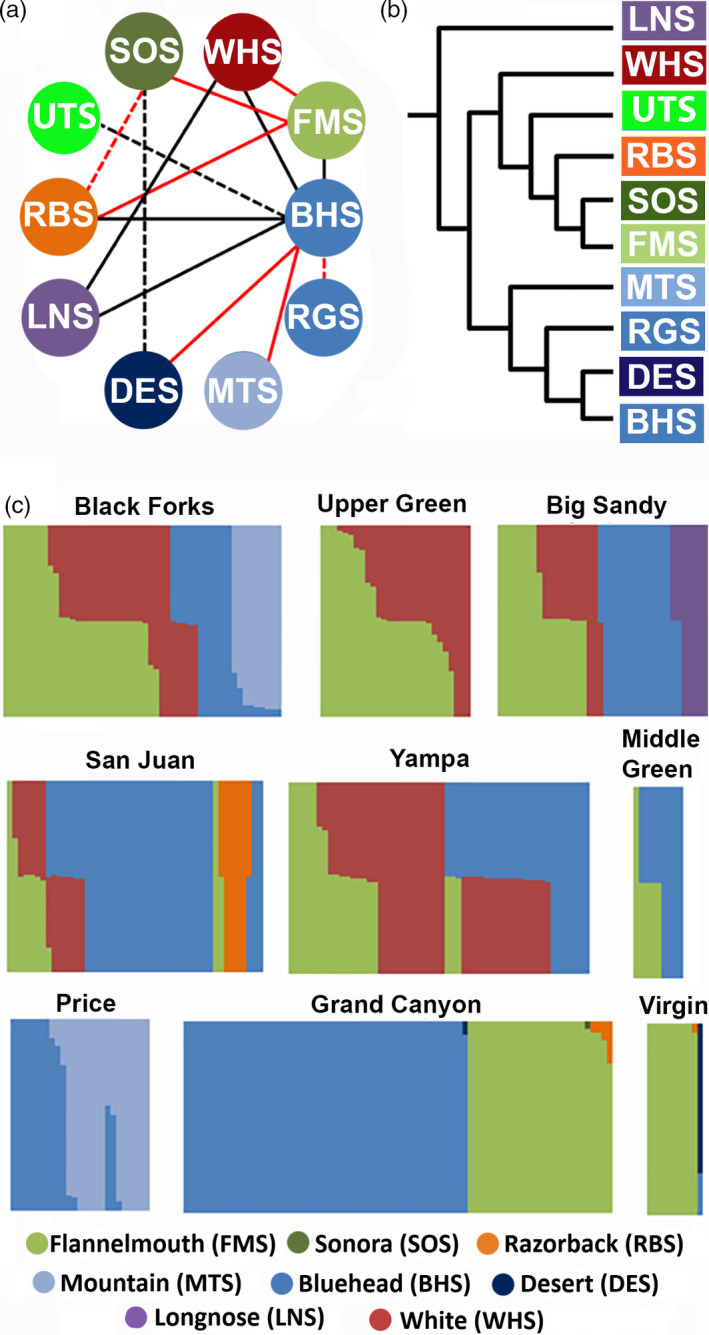
(a) Network depicting crosses among study species. Solid lines = those recorded in this study; dashed = previous studies (Clarkson & Minckley, 1988). Red lines = introgression; black lines = hybridization without introgression. Species abbreviations as in Appendix A and are colored by subgenus or species (shades of green and red = *Catostomus*; blue = *Pantosteus*; orange = *Xyrauchen*; purple = Longnose Sucker). (b) Species relationships, as depicted in Bangs et al. (2018). (c) Bayesian clustering plots by region for those populations with admixed ancestry (343 samples). These plots do not include the 66 samples used as reference, which can be found in Figure S2

One sample, collected in the Navajo River, was assigned to three species: Bluehead Sucker (*q* = 0.50), White Sucker (*q* = 0.37), and Flannelmouth Sucker (*q* = 0.13). Since this sample has assignment to more than two species, it could not be used for calculating a hybrid index or for assignment to a hybrid category in NewHybrids. All other samples were assigned to two clusters at most and thus were utilized for hybrid analyses. However, the high interspecific heterozygosity value for this sample indicates that it was a first‐generation cross of a Bluehead Sucker and with a White Sucker × Flannelmouth Sucker hybrid. (Figure 2c).

Average pairwise genetic distances between species, as computed in phylip, are presented in Table 2. Introgression was detected only between species separated by genetic distances < 2%. On the other hand, hybridization without introgression was only recorded in species pairs exhibiting genetic distances between 2.2% and 2.9%.

**Table 2:** Average pairwise genetic distances calculated between species of *Catostomus* (Pisces: Catostomidae) from the complete sequence alignment of all 14,007 loci used in Bangs et al. (2018)

Taxa	BHS	DES	RGS	MTS	FMS	SOS	RBS	UTS	WTS	LNS
BHS	X									
DES	0.3%	X								
RGS	0.6%	0.6%	X							
MTS	1.2%	1.3%	1.5%	X						
FMS	2.2%	2.3%	2.4%	2.4%	X					
SOS	2.3%	2.4%	2.6%	2.5%	0.3%	X				
RBS	2.5%	2.6%	2.6%	2.7%	0.6%	0.6%	X			
UTS	2.2%	2.3%	2.3%	2.3%	1.0%	1.1%	1.2%	X		
WTS	2.2%	2.4%	2.3%	2.3%	1.6%	1.8%	1.9%	1.6%	X	
LNS	2.8%	3.0%	2.9%	3.0%	2.9%	3.0%	3.2%	2.9%	2.9%	X

Note

Distances were calculated using the default F84 model in dnadist, as implemented in PHYLIP (Felsenstein, 1993). Cells highlighted in color represent: blue = subgenus *Pantosteus*; green = subgenus *Catostomu*s; orange = genus *Xyrauchen*; and red = an introduced species.

Abbreviations: BHS, Bluehead Sucker; DES, Desert Sucker; FMS, Flannelmouth Sucker; LNS, Longnose Sucker; MTS, Mountain Sucker; RBS, Razorback Sucker; RGS, Rio Grande Sucker; SOS, Sonoran Sucker; UTS, Utah Sucker; WTS, White Sucker.

### 3.2 Hybridization with invasive species

The two introduced species differ in their distributions, with White Sucker widespread but Longnose Sucker restricted to the Big Sandy River (WY). White Sucker × Flannelmouth Sucker hybrids occurred in all regions in which both parental species are common. This included the three northern‐most regions in Wyoming, Yampa River, and the Navajo River tributary of the San Juan River (Figure 1a). White Sucker × Bluehead sucker were also found in the same locations, with the exception of the Upper Green River region where Bluehead Sucker is less common (Figure 2c).

All White × Bluehead sucker hybrids (*N* = 29; Figure 4b) reflected high interspecific heterozygosity and hybrid indexes of ~ 0.50 and were assigned by newhybrids as F1. White × Flannelmouth sucker (*N* = 68) were identified by *q*‐scores (structure: Figure 2c) and hybrid indices (introgress: Figure 4a). The majority of ~ 70% (*N* = 46) were assigned by newhybrids as F1, with the remainder as F2 (*N* = 3), first‐generation backcross (Bx) to White (*N* = 6) or Flannelmouth (*N* = 8) sucker, or second‐generation Bx to Flannelmouth Sucker (*N* = 2). Three were undetermined. However, most regions had only F1 hybrids and first‐generation backcrosses to Flannelmouth Sucker. Backcrosses (F2) were restricted to part of the basin [Figure 4j: Upper Green River and Muddy Creek of the Yampa River]. Similarly, first‐generation White Sucker backcrosses were found only in the Upper Green (Figure 4j) and Ham's Fork of Blacks Fork (Figure 4h). Second‐generation backcrosses with Flannelmouth Sucker, as well as undetermined hybrid classes, only occurred in the Upper Green (Figure 4j).

Longnose Sucker hybridized with both invasive White Sucker (Figure 4c) and native Bluehead Sucker in the Big Sandy River (Figure 4f). All three were assigned a *q*‐score and hybrid index of 0.50 (Longnose, Bluehead, or White sucker). High interspecific heterozygosity values and output from newhybrids also underscored their status as F1 hybrids.

### 3.3 Hybridization between endemic species

Hybrids between the two widespread species, Flannelmouth and Bluehead sucker, were found in two areas: The Yampa River, and throughout the Middle Green River region, to include the White River and the mainstem Green River between above its confluence with the White River (Figures 1b and 2c). All seven individuals reflected hybrid indices of ~0.50 with high interspecific heterozygosity (Figure 4d) and were categorized as F1 by Newhybrids. These assignments were consistent with *q*‐scores that approximated 0.50 (Figure 2c).

**Figure 3:**
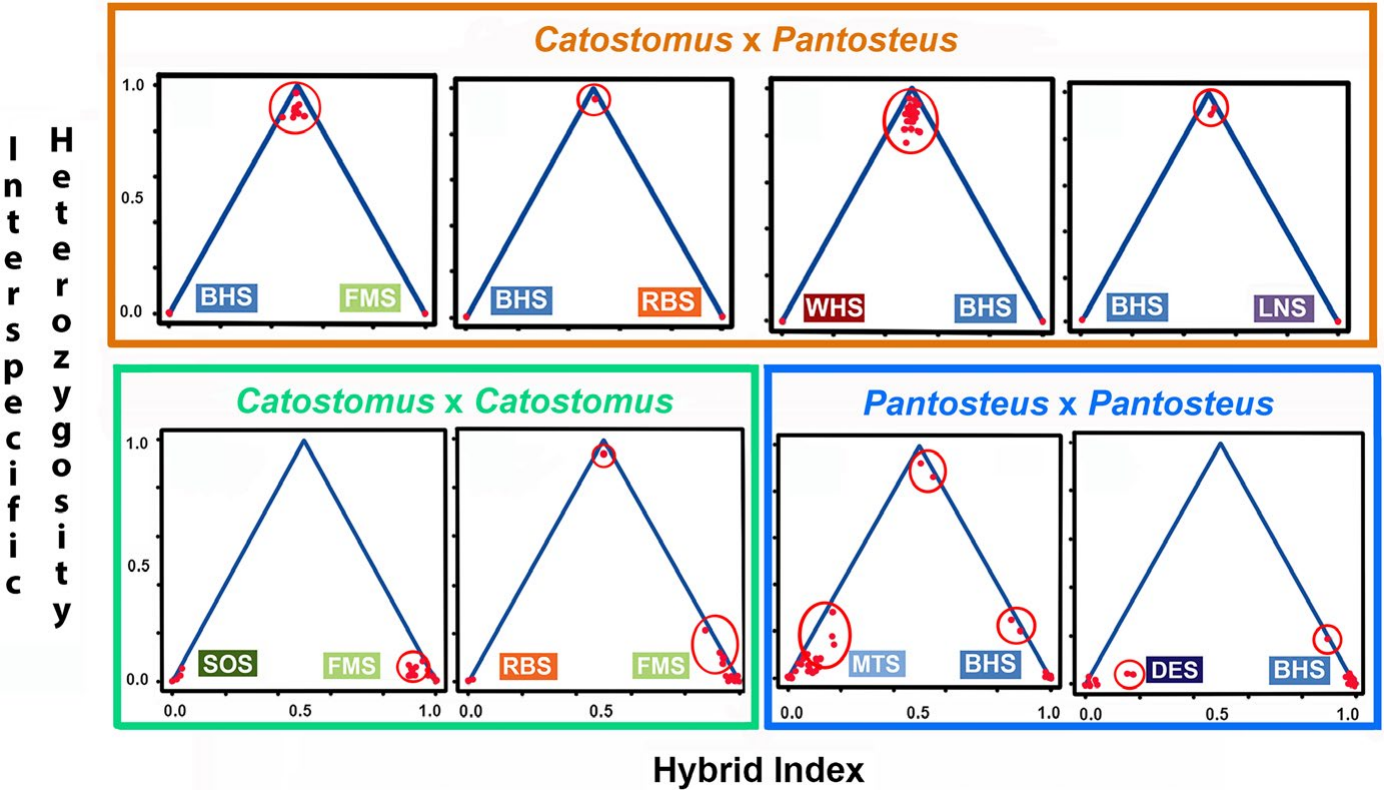
Triangle plots comparing hybrid indices versus interspecific heterozygosities for species found within subgenera of *Catostomus*. Top row, orange box: species in *Catostomus* (green, red, orange, and purple) versus species in subgenus *Pantosteus* (blue), where FMS, Flannelmouth Sucker; RBS, Razorback Sucker; WHS, White Sucker; LNS, Longnose Sucker; BHS, Bluehead Sucker. Bottom row, green box (left): Species within subgenus *Catostomus*, with SOS = Sonoran Sucker. Bottom row, blue box (right): Species within subgenus *Pantosteus*, with MTS, Mountain Sucker; DES, Desert Sucker. Red circles indicate hybrids, with those at the top of the triangle being F1 hybrids and those lower in the triangle indicating various levels of introgression

**Figure 4:**
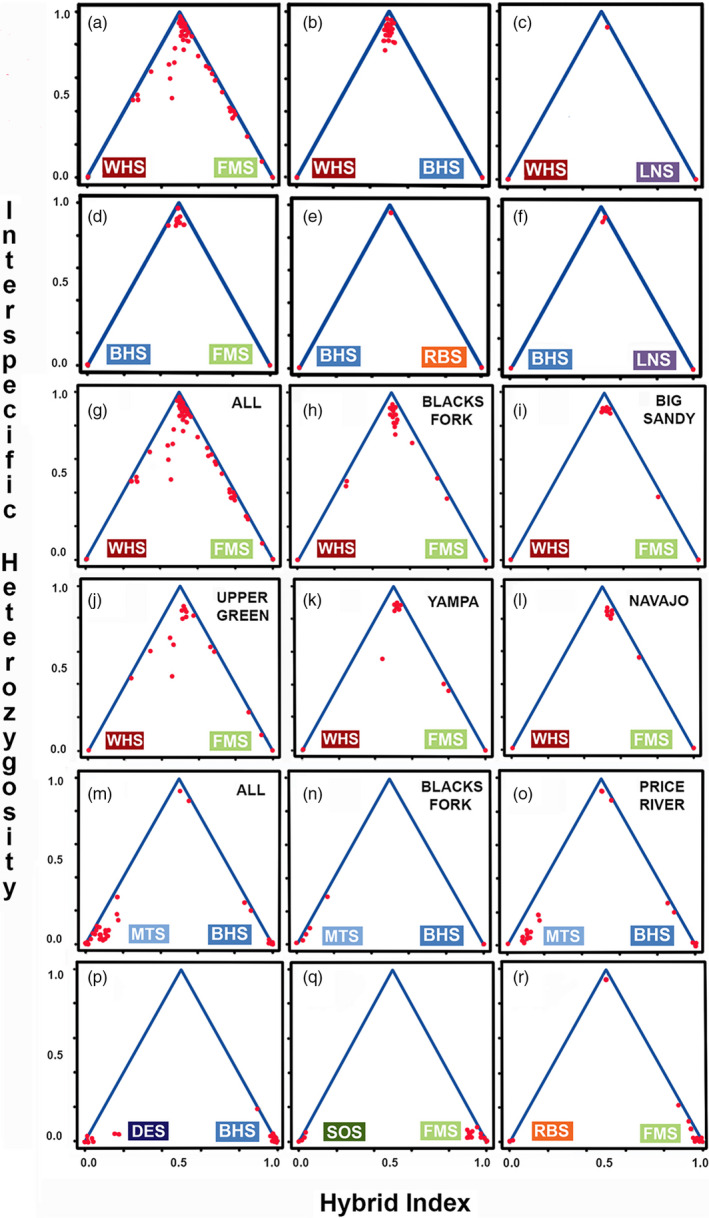
(Row 1): Triangle plots depicting hybrid indices versus interspecific heterozygosities for species of *Catostomus,* to include introduced White (WHS) and Longnose (LNS) sucker. Crosses include (a) White × Flannelmouth sucker (WHS × FMS); (b) White × Bluehead sucker (WHS × BHS); (c) White × Longnose sucker (WHS × LNS). (Row 2): Triangle plots depicting hybrid indices versus interspecific heterozygosities for Bluehead Sucker and other *Catostomus* external to the subgenus *Pantosteus*: (d) Bluehead × Flannelmouth (BHS × FMS) sucker; (E) Bluehead × Razorback (BHS × RBS) sucker; (f) Bluehead × Longnose (BHS × LNS) sucker. (Rows 3 and 4): Triangle plots depicting hybrid indices versus interspecific heterozygosities by location for White × Flannelmouth sucker: (g‐l). (Rows 5 and 6): Triangle plots depicting hybrid indices versus interspecific heterozygosities for species within the same subgenus: (m‐o) Subgenus *Pantosteus* by location*,* Mountain × Bluehead sucker (MTS × BHS); (P) Subgenus *Pantosteus*, Desert × Bluehead sucker (DES × BHS); (q) Subgenus *Catostomus*, Sonora × Flannelmouth sucker (SOS × FMS); (r) Subgenus *Catostomus*, Razorback × Flannelmouth sucker (RBS × FMS). BHS, Bluehead Sucker; FMS, Flannelmouth Sucker; LNS, Longnose Sucker; RBS, Razorback Sucker; WHS, White Sucker

Hybrids involving the widespread, but rare Razorback Sucker were only found in the mainstem San Juan River near its confluence with the Colorado River. These included one F1‐hybrid with Bluehead Sucker (Figure 4e) and one F1‐hybrid with Flannelmouth Sucker (Figure 4r). The F1‐classification was consistent across all three analyses. Introgressed hybrids between Razorback and Flannelmouth sucker were found in the southwestern area, the Virgin River and Grand Canyon (Douglas & Marsh, 1998). All are seemingly high‐level backcrosses to Flannelmouth Sucker, given that newhybrids failed to assign them to any hybrid category. In addition, contained *q*‐scores and hybrid indexes > 0.75 for Flannelmouth Sucker (Figures 2c and 3r).

Other hybrids involved the two endemic species from the Lower Colorado River basin: A Bluehead × Desert sucker hybrid and a Flannelmouth × Sonora sucker hybrid were found in Grand Canyon, and one Bluehead × Desert sucker hybrid in the Virgin River. These assignments were consistent across both Bayesian clustering (Figure 2c) and hybrid index (Figure 4p,q), but their low interspecific heterozygosity (as expected from historic hybridization) precluded their assignment to any hybrid category. Flannelmouth Sucker from the Virgin River assigned completely to the Flannelmouth Sucker cluster (Figure 2c), but showed significant hybrid indices based on a 95% confidence interval indicating some level of historic introgression (Figure 4q).

Mountain Sucker, restricted to higher elevation areas in the northern half of the basin, reflected hybrids in two northwestern areas: Bluehead × Mountain sucker hybrids in the Blacks Fork region (Figure 4n), and the Price River (Figure 4o). Interestingly, all Price River individuals were field‐identified as Mountain Sucker, whereas one (of nine) from Blacks Fork was field‐identified as Bluehead Sucker. Of the nine hybrids in Blacks Fork, two were classified as a first‐generation backcross to Mountain Sucker, with the remaining seven as later‐generation backcrosses, based on elevated assignments to Mountain Sucker in both Bayesian clustering (Figure 2c) and hybrid index (Figure 4n). Of the eight hybrids in the Price River region, five were caught in the Price River and three in the White River (Figure 4o). newhybrids classified two from the White River as F1‐hybrids, one from the Price River as first‐generation backcross to Bluehead Sucker, while the remaining five were undetermined and presumably higher‐level backcrosses into either Bluehead Sucker or Mountain Sucker. Unlike Blacks Fork, the Price River also had several field‐identified Mountain Sucker that were assigned as such by Bayesian clustering (Figure 2c).

## 4 DISCUSSION

A more formal exploration of hybridization, and of reticulated evolution in general, has been promoted by contemporary advancements in sequencing technology, with more expansive datasets developed as a consequence (Eaton & Ree, 2013; Kane et al., 2009). Given this, a much less cumbersome view of introgressive hybridization has emerged, one that promotes instead the maintenance of semipermeable species boundaries, the consequences of which have impacted evolutionary thought (Nosil, Funk, & Ortiz‐Barrientos, 2009; Harrison, 2012; Michel et al., 2010). For example, we now understand that introgression can occur without subsequent dismantling of species boundaries (Fontaine et al., 2015), and likewise, with a rather precise transmission of adaptive traits (Dasmahapatra et al., 2012; Nadeau et al., 2012). This has reshaped both our view of speciation, as well as the manner by which reproductive isolation can evolve in the face of contemporary and historic hybridization (Edmands, 2002). It also broadens our concept of how admixture can facilitate adaptation. For example, a gene region that controls color pattern expression in *Heliconius* butterflies has been identified as part of a chromosomal inversion that is transferred intact during admixture, allowing for color patterns to be switched (Edelman et al., 2019). How these insights affect conservation and management of wild species is still evolving, especially when the perceived negative impacts of invasive hybridization are superimposed onto a complex system with a long history of reticulate evolution among native species. Here, we build on our previous work to demonstrate how genomic tools can not only resolve this complexity, but also promote new perspective that can facilitate the adaptive management of species that are of conservation concern.

Catostomid fishes are a good system to gauge the manner by which reproductive isolation, or lack thereof, has evolved for several reasons. They display (a) an historic tendency to hybridize (Buth et al., 1992; Dowling et al., 2016; Hubbs et al., 1943; Nelson, 1968; Tranah & May, 2006); (b) A deep, chaotic history of isolation and secondary contact, as driven by the geology of western North America (Smith et al., 2013); and (c) conservation concerns that stem in large part from hybridization with invasive congeners. However, and despite these caveats, their introgression remains relatively enigmatic, particularly across geographic and temporal scales, and this clearly impacts their management.

The same reasons that make catostomid fishes in the Colorado River Basin a good system to study hybridization also makes them a challenging group to tackle. For example, attempts to quantify historic introgression that occurred millions of years ago may be misled by contemporary hybridization spurred on by habitat modification and introduced species, and in this same regard, ongoing hybridization and introgression might be blurred by historic introgression. Also, studying hybridization on a local scale might be fallacious by failure to include species from neighboring regions, or result in conclusions accurate locally but that are inaccurate if extrapolated to a larger scale. In contrast, studying hybridization on a basin‐wide scale can fail to quantify the extent at which ingression and hybridization are occurring in individual populations and management units. Thus, the goal of this study is not to answer all of these questions at once but instead build on the recent literature that has already quantified historic hybridization and reticulate evolution (Bangs et al., 2018), explored species delimitation (Bangs et al., 2020), and explored the extent of hybridization and introgression on a local scale (Mandeville et al., 2015; Mandeville et al., 2017), by examining hybridization on a basin‐wide scale and then evaluating results from these recent genomic studies in a comparative framework to better understand and disentangle the history of hybridization in the system.

Our case study documents all possible patterns of hybridization that have occurred across an array of hybrid crosses involving 10 species in the Colorado River Basin (Figures 1b and 2). Our results highlight a level of reproductive isolation that increases with phylogenetic distance, as well as a recognition of the variability in the outcomes of hybridization, as displayed across an entire basin. These data provide insights into the evolution of reproductive isolation, a consequence that can not only inform conservation, but also predict potential patterns of admixture as rivers inevitably dwindle due to drought and anthropogenic water use (Cayan et al., 2010).

### 4.1 Reproductive isolation as a component of phylogenetic distance

Reproductive isolation is expected to increase with phylogenetic divergence, especially if phenotypic differences promote ecological specialization among taxa (Coyne & Orr, 2004). Ecological divergence, an important driver of reproductive isolation (Funk, Nosil, & Etges, 2006), has been suggested as such in *Catostomus* despite repeated occurrences of hybridization and introgression (Mandeville et al., 2015). Here, we find that while hybridization transects all phylogenetic levels within the genus, barriers to introgression increase with phylogenetic distance, particularly between those subgenera that display different life histories and habitat preferences.

The phylogeny of *Catostomus* includes two subgenera (*Catostomus* and *Pantosteus* as described Smith et al., 2013) with Longnose Sucker as sister to the two subgenera (Figure 2b). These subgenera were described morphologically (Smith et al., 2013) and confirmed with mitochondrial (Unmack et al., 2014) and genomic (ddRAD) data (Bangs et al., 2018) and represent two ecologically specialized types (i.e., mainstem river versus mountain stream specialist). Crosses between subgenera (i.e., Flannelmouth × Bluehead sucker, White × Bluehead sucker, Razorback × Bluehead sucker; Figure 3 top row) did not exhibit any introgression, as did crosses with Longnose Sucker (Longnose × White sucker and Longnose × Bluehead sucker Figure 4c,f). In comparison, all four crosses within subgenera did reflect introgression (Figure 3 bottom row). This includes Flannelmouth × Razorback sucker, each currently within a different genus, but with nuclear (Bangs et al., 2018) and mitochondrial (Chen & Mayden, 2012) data placing both within the subgenus *Catostomus*. The nestling of Razorback Sucker within the subgenus *Catostomus* based on molecular data is incongruent with morphological data and can be attributed to it representing a third ecotype adapted to lotic systems. Lake‐dwelling suckers, including *Chasmistes* and *Deltistes*, have been placed outside of the *Catostomus*/*Pantosteus* grouping, but may have split as early as 8–10 mya based on the fossil record, which is more recent than the estimated 23.9 mya *Catostomus*/*Pantosteus* split (Unmack et al., 2014). Lake suckers also show evidence of multiple hybridization events throughout their evolutionary history, often associated with droughts, and may have an evolutionary benefit of parasite avoidance (Smith et al., 2018). Thus, for simplicity sake, we included Razorback Sucker in the subgenus *Catostomus* given the overall genetic similarity (Table 2).

This pattern of introgression within subgenera, and a lack thereof between them, remains consistent even when expanded to include other hybrid crosses and drainages: the Bonneville Basin (Bangs et al., 2017; Utah × Bluehead sucker), the Lower Colorado River Basin (Desert × Sonora sucker, Clarkson & Minckley, 1988; Sonora × Razorback sucker), and the Little Colorado River (Bangs et al., 2020; Bluehead × Rio Grande sucker, and Flannelmouth × Sonora sucker). All crosses among‐subgenera reflect introgression, either contemporaneous or historic, whereas it is absent in all crosses between subgenera (Figure 2a). The sharp differences in patterns of introgression within and between subgenera relate to a breadth and depth of reproductive isolation. This increase in reproductive isolation may simply be driven by the degree of phylogenetic divergence (~24 mya between subgenera and ~14 mya within; Unmack et al., 2014) leading to increase genetic incompatibility.

This pattern may also be ecologically driven. For example, species within the subgenus *Pantosteus* prefer cooler, higher elevation habitats as compared to those within the subgenus *Catostomus* (Sigler & Miller, 1963). In addition, *Pantosteus* also demonstrates a series of specialized morphological adaptions that facilitate the scraping of diatoms and biofilm from the substrates of high‐velocity streams (Smith, 1966). Thus, ecological specializations may also promote reduced introgression, in that the fitness of hybrids is depressed in either parental environment. As noted above, the Razorback Sucker is ecologically specialized for lake or large bodies of water; however, they can readily introgress with Flannelmouth Sucker which goes against the idea of ecological specialization playing a major role in levels of isolation. Still, introgression may have an evolutionary benefit for in Razorback Sucker as mechanism to reduce parasite risk (Smith et al., 2018).

Regardless of the reason, the pattern of reduce introgression across subgenera is pronounced in this study and has previously been suggested in population‐level studies of three species (Mandeville et al., 2015) that focused on quantifying levels of introgression at the local scale. Our study documents that this trend is maintained at a broad geographic scale and across a wider breath of species.

### 4.2 Invasive species hybridize with native species ‐ but without a hybrid bridge

A non‐native species, White Sucker, has been introduced throughout the Upper Green, Yampa, and San Juan rivers (Holden, 1991; Sublette, Hatch, & Sublette, 1990) and now hybridizes with both Flannelmouth and Bluehead sucker (Douglas & Douglas, 2010; Holden & Stalnaker, 1975; McDonald et al., 2008; Quist, Bower, Hubert, Parchman, & McDonald, 2009). It has been argued (McDonald et al., 2008) that introduced White Sucker now threatens the reproductive isolation of both Flannelmouth and Bluehead sucker by acting as a “hybrid bridge” that can serve to connect the two endemic species. However, our results refute this hypothesis by demonstrating that Flannelmouth and Bluehead sucker hybridize in the absence of White Sucker. Furthermore, introgression of Flannelmouth occurred with White sucker, but not Bluehead Sucker.

Seven F1 hybrids were found between Flannelmouth × Bluehead sucker, four of which occurred in the Middle Green River region where White Sucker is absent, or at best uncommon. All were found in the Green River above its confluence with the White River, as well as White and Yampa rivers themselves (Figure 1a). This area on the Green River is impacted by Flaming Gorge Dam (Figure 1b), which altered downstream habitat, reshuffling the distribution and abundance of native suckers, and consequently disrupted reproductive isolation of endemic species in the Middle Green, White, and Yampa rivers. These habitat modifications also promoted the distribution of White Sucker and its hybrids (Holden & Stalnaker, 1975).

If an introduced species (i.e., White Sucker) does in fact serve as a hybrid bridge, one would also expect hybrids to be found with DNA from all three species. Yet, only one such individual was found (i.e., Navajo River in the San Juan River drainage), despite the presence Flannelmouth × White sucker and Bluehead × White sucker in other areas (Figure 2c). Thus, three‐way crosses are not only extremely rare, but also restricted to particular geographic regions where introgression between Flannelmouth and White sucker is more common, and where Bluehead Sucker is abundant. This is also reflected in a recent study confirming the presence of admixed individuals between the three species in Muddy Creek (Yampa River), but not the Big Sandy, even though Flannelmouth × White sucker and Bluehead × White sucker are present in both (Mandeville et al., 2015). Three‐way hybrids were found to be 50% ancestral to Bluehead Sucker, and may thus represent a first‐generation cross between Bluehead Sucker and Flannelmouth × White sucker hybrids. This also fits well with our previous argument indicating a lack of introgression across subgenera.

In either case, introgression was not detected for any Bluehead Sucker hybrids, except within the subgenus *Pantosteus*. Thus, admixture with Flannelmouth Sucker, White Sucker, and Flannelmouth × White sucker hybrids is not a threat to the genetic integrity of Bluehead Sucker and does not to contribute to a hybrid swarm (per McDonald et al., 2008). However, it does represent a loss of reproductive effort, and management should therefore be aimed at this aspect.

In comparison with White Sucker, the impact of Longnose Sucker, a second introduced species, has been minimal. Its presence was recorded only in the Big Sandy River, where it hybridized with both native Bluehead and introduced White sucker. We found no evidence for introgression, a result not surprising given their deep phylogenetic divergence (~27.9 mya; Unmack et al., 2014; Table 2). In addition, other studies in the Big Sandy River found but a few Bluehead × Longnose sucker hybrids, all of which were presumably F1s (Mandeville et al., 2015).

### 4.3 Introgression increases with habitat alteration

Introgression between native Flannelmouth Sucker and introduced White Sucker can, however, be construed as a threat to the genetic integrity of Flannelmouth Sucker, already listed as a “species of concern” throughout its range. Yet, this threat varies by region. Some (i.e., Upper Green River and Blacks Fork regions) reflect greater levels of introgression than do others (Figure 4h and 4j). In the Yampa River region, F2 and Bx hybrids were detected, but solely from Muddy Creek, a drainage where suckers were previously impacted by extensive introgression (Mandeville et al., 2015; McDonald et al., 2008). Despite the presence of several F1 hybrids, no evidence for introgression was found in the mainstem Yampa and Little Snake rivers, a result consistent with that of Douglas and Douglas (2010). The Big Sandy River contained only one Bx and several F1 hybrids (Figure 4i), again juxtaposing with the limited introgression found in this region (Mandeville et al., 2015).

The extent of introgression between species documented in our analyses can be attributed to habitat alterations. All sites with obvious introgression are found in Wyoming, an area of the Upper Colorado River basin characterized by anthropogenic impacts. These include (a) dumping of industrial pollutants and raw effluent in the 1940s (Bosley, 1960); (b) development of Flaming Gore and Fontenelle dams in the early 1960s; (c) extensive rotenone treatment to remove “trash” fish in 1962; and (d) introduction of numerous invasive fishes (Holden, 1991). Collectively, these actions reduced native fish densities, particularly suckers, as well as greatly modified the habitat of the region (Quartarone, 1995; Wiley, 2008). The probability is thus elevated that habitats in this region have been homogenized, reproductive behaviors impacted, and hybridization promoted, such that hybrid survival is facilitated. This is especially apparent in the Upper Green River, where the brunt of these impacts occurred, with Bluehead and Razorback sucker now rare or absent (Wiley, 2008). These regions also manifest the greatest levels of introgression between Flannelmouth and White sucker (Figure 4j).

### 4.4 Contemporary hybrids between native species

Along with Bluehead × Flannelmouth hybrids (mentioned above), several other contemporary hybrids were detected between native species, to include Bluehead × Mountain sucker as well as hybrids with Razorback Sucker.

Bluehead and Mountain sucker share a long history of introgression in the Colorado and Bonneville river basins and also hybridize in the Little Sandy River (Mandeville et al., 2015). However, our range‐wide assessment found introgressive hybridization between these species in Blacks Fork (Figure 4n) and Price River (Figure 4o), as well as two F1 hybrids in the Price River. These data emphasize how contemporaneous the hybridization between these species has been and, in turn, reflects not only habitat alterations in the Upper Green River (WY) but also the introduction of Mountain Sucker from the Bonneville Basin into the Price River (Sigler & Miller, 1963).

Razorback Sucker was historically distributed throughout the entire Colorado River Basin, but has experienced drastic declines (Douglas & Marsh, 1998; Minckley, 1983), leading to its listing as an endangered species (US Fish and Wildlife Service, 1991). Declines are attributed to habitat alteration, to include development of dams that not only disrupt recruitment but increase opportunities for hybridization with Flannelmouth Sucker (Buth, Murphy, & Ulmer, 1987). Several hybrids involving Razorback Sucker in the Grand Canyon and Virgin River were high‐level backcrosses with Flannelmouth Sucker, as would be expected from an initial hybridization followed by several generations of backcrossing (Figures 2c and 4r).

Similar results were found in a four‐year mark–recapture study of Flannelmouth Sucker in Grand Canyon (Douglas & Marsh, 1998), where the hybrid population was estimated to be ~ 30 (8–136). Over the four‐year study, 41 morphologically diagnosed Flannelmouth × Razorback hybrids were not only captured but subsequently recaptured 60 times. Twelve of these were evaluated using molecular markers (T.E. Dowling, pers. comm.), and eight were determined to be of hybrid origin with Flannelmouth Sucker (but none designated as F1).

In addition, two F1 Razorback hybrids were also found in the San Juan River (Figure 1b). One was with Flannelmouth Sucker, which has been known to hybridize, and the second was an F1 cross with Bluehead Sucker sampled from the mainstem San Juan River that, to our knowledge, has not been previously documented. Finding a couple of these hybrids may not represent much of a loss of natural recruitment and reproductive output, both of which have been drastically reduced in Razorback Sucker (Minckley, 1995). However, these hybrids may be important to note given that stocking programs to rehabilitate Razorback Sucker were initiated in 1991, with several populations subsequently augmented to include the San Juan River (Dowling, Minckley, & Marsh, 1996; Dowling et al., 2014; Minckley, 1995). While two hybrids might not represent much of a threat to this program, it does underscore that hybridization is occurring. Importantly, these documented instances may not represent the true level of hybridization and introgression in this area, since both were random samples sent to us for analysis and do not represent a true population‐level assessment of suckers in this area.

### 4.5 Historic hybridization between Lower and Upper Basin species

An interesting result in our analyses was an echo of historic hybridization between species currently allopatric. Sonoran and Desert sucker, found below Grand Canyon in the Lower Colorado River Basin (Figure 1a), are ecologically equivalent to Flannelmouth and Bluehead sucker from the Upper Colorado Basin. One Flannelmouth × Sonora sucker and one Bluehead × Desert sucker hybrid were found in Grand Canyon, a conduit between Upper and Lower Colorado River basins. Hybridization between these species has been suggested due to elevated morphological variation in both Grand Canyon and Virgin River (Minckley, 1980), as well as the presence of conspecific mitochondrial haplotypes in the Upper Colorado River Basin (Douglas et al., 2003; Douglas & Douglas, 2010; Hopken et al., 2013). Historic contact could have occurred as climate changed during the Mid Holocene, when a prolonged drought during the Hypsithermal drastically reduced flow in the Colorado River. This may have forced Upper Basin species into the Lower Basin as flows diminished, thus promoting contact between sister species (per Douglas et al., 2003).

Introgression between these sister species was also found in the Virgin River, an area in close proximity to Grand Canyon (Figure 1a). The Virgin River has a unique fish assemblage, due to the presence of both Flannelmouth Sucker (native to the Upper Basin) and Desert Sucker (native to the Lower Basin). However, neither Sonora Sucker nor Bluehead Sucker are found there. Yet, highly significant introgression was found between sister species, suggesting the potential hybrid origin of co‐occurring species therein. Bayesian clustering did assign the Flannelmouth Sucker from the Virgin River to the Flannelmouth Sucker cluster, but also recognized a significant hybrid index with Sonora Sucker. On the other hand, Desert Sucker from the Virgin River showed significant introgression with Bluehead Sucker in both Bayesian clustering and hybrid indices, based on a single sample. Additional samples for Flannelmouth and Desert sucker from the Virgin River are needed before the effects of introgression can be fully elucidated in this region.

### 4.6 Adding to the growing body of genomic work and its implications on management

We hope that this work along with the growing body of conservation genomic literature on *Catostomus* can lay out a blueprint on how to disentangle the complexity of hybridization and introgression. Hybridization in the genus has been suggested to occur throughout their history leading to historic introgression events that resulted in discords between mitochondrial and morphological phylogenies that hinder species delimitation and studies of contemporary hybridization. On top of this, contemporary hybridization can occur between both endemic species as well as with introduced species making it harder to decipher anthropogenic and natural processes. In order to resolve these issues, there is a need to (1) examine historic introgression in a phylogenomic framework to resolve discords in previous mitochondrial and morphological phylogenies, then (2) use this framework to examine species delimitation, (3) examine contemporary hybridization in the absence of introduce species to understand natural hybridization and introgression processes, and then (4) examine the correlation with anthropogenic impacts, to include introduced species and habitat change, on rates and patterns of hybridization and introgression.

Points 1 and 2 have been addressed in recent genomic studies. Bangs et al. (2018). used ddRAD to examine the phylogeny of the genus and showed that historic introgression had occurred and in turn explains discordance between morphological and mitochondrial phylogenies. This allowed Bangs et al. (2020) to examine species delimitation models using both phylogenomics and population genomic methods, which outlined conservation units in Flannelmouth and Bluehead suckers, confirmed the species level split of Bluehead Sucker between the Bonneville and Colorado River (the former is likely to be listed due to drastically declining populations), and examined the evolution of an endangered species, Zuni Bluehead Sucker, recently federally listed under the U.S. Endangered Species Act (ESA). The latter has an extensive history of admixture that added complexity to an ongoing debate about the geographic range of the species and highlighted the need to thoroughly evaluate sucker populations in areas previously understudied in the Little Colorado River drainage. Combined, the studies also encompass the geographic extent of these species and allowed for us to examine contemporary hybridization and introgression, as done herein, and tackle points 3 and 4 above.

Here, we show that hybridization occurs between endemic species in the Upper Colorado River Basin, even in the absence of introduced species and that introgression is limited with increasing phylogenetic distance, either due to ecological specialization or genetic incompatibilities. While our study could not quantify the exact levels of introgression in each population, it does corroborate the results of previous population‐level genomic studies (Mandeville et al., 2015, 2017) that suggested introgression is rare and might be limited to certain crosses, populations or areas with increased habitat modification. This pattern is maintained by our analyses at a larger geographic scale (basin‐wide) and a broader taxonomic spectrum to include all 10 species that occur in the basin.

Due to minimal rates of introgression found in most locations and the rarity of hybrids with ancestry of more than two species across the basin, as well as in a focused population study in the upper reaches of the Upper Colorado River Basin (Mandeville et al., 2017), the capacity for White Sucker to serve as a “hybrid bridge” between native species is negligible, and the implication that multiple species will potentially collapse into a “mutt sucker” (per McDonald et al., 2008) is improbable. The concern of a complete collapse of reproductive isolation to the point of a multispecies hybrid swarm is unlikely. Management efforts should therefore not focus on the removal of hybrids, an arduous endeavor at best with marginal effects, but instead be directed toward habitat restoration, since hybridization and introgression appear to be promoted by habitat disturbance.

These studies demonstrate how multiple conservation genomic studies can work in tandem to provide synergistic insights into complex and challenging systems. Future work is still needed on understanding why these patterns of introgression have occurred, with particular interest on quantifying what factors of habitat disturbance lead to increased introgression, the fitness impacts of different hybrid genotypes, the uniformity of introgression, or lack thereof, across genomic clines (i.e., super invasive alleles), and detecting loci that might increase genetic incompatibles, all of which can play an important role in conservation decisions (i.e., see Arnold, 2016).

## 5 CONCLUSIONS

While hybridization is increasingly recognized as a common evolutionary phenomenon among fishes, our case study of catostomid fishes from the Colorado River basin suggests introgression seemingly decreases with phylogenetic distance and may be driven by ecological specializations that separate subgenera. Furthermore, introgression between a native and introduced species has increased concomitant with habitat disturbance (also suggested by Mandeville et al., 2015). However, the capacity of an introduced species to serve as a “hybrid bridge” between native species, as suggested for White Sucker (per McDonald et al., 2008), is negligible at a larger scale, particularly given the extreme influence of habitat alterations in promoting breakdown of reproductive isolation among native species (per Middle Green, Yampa, and White rivers). Based on our analyses, the implication that multiple species will potentially collapse into a “mutt sucker” (McDonald et al., 2008) is improbable, due to minimal rates of introgression found in most locations coupled with the increased level of reproductive isolation concomitant with phylogenetic divergence.

The presence of historic admixture between native species also provides an example of how species boundaries can be maintained, even in the presence of anthropogenically induced introgression. This study examines hybridization and introgression across an entire freshwater basin, to include all native or introduced catostomids in the system. Understanding the existing patterns of hybridization and reproductive isolation across this diverse range of species provides a baseline necessary to disentangle the long history of hybridization among fishes in western North America. These data, in turn, will promote region‐wide adaptive management and conservation.

## CONFLICT OF INTEREST

None declared.

## Supporting information

Figures S1‐S2

## Data Availability

Data for this study will be available at the Dryad Digital Repository once the manuscript is accepted for publication.

## Appendix A

### Sample sites and number of samples based on field identification. BHS, Bluehead Sucker; DES, Desert Sucker; FMS, Flannelmouth Sucker; HYB, Hybrid; LNS, Longnose Sucker; MTS, Mountain Sucker; RBS, Razorback Sucker; RGS, Rio Grande Sucker; SOS, Sonora Sucker; UTS, Utah Sucker; WHS, White Sucker

**Table:**

Region	Location	State	*N*	FMS	BHS	WHS	MTS	LNS	RBS	DES	SOS	RGS	UTS	HYB
Upper Green River	Horse Creek	WY	4											4
	Cottonwood Creek	WY	10	4										6
	Bitter Creek	WY	7	3										4
	Flaming Gorge Dam	WY	6	2		2								2
Big Sandy River	Big Sandy River	WY	25	4	6		1	5						9
	Little Sandy River	WY	20	1	6		1							12
Blacks Fork	Hams Fork	WY	13	4										9
	Muddy Creek	WY	9	3										6
	Blacks Fork	WY	20	2	3		7							8
	Henrys Fork	WY	8	3	2		1							2
Yampa River	Little Snake River	WY	21	2	4	3								12
	Yampa River	CO	39	5	5	5	1							23
Middle Green River	Green River	UT	7		4									3
	White River	UT	4	1	2									1
Price River	Price River	UT	15		8		7							
	White River	UT	10				10							
San Juan River	Navajo River	NM	17	2	3									12
	Arch Canyon	UT	20		20									
	San Juan River	UT	10	2	2				4					2
Grand Canyon	Havasu Creek	AZ	10		10									
	Matkatamiba Canyon	AZ	15		15									
	Kanab Creek	AZ	12	10	2									
	Shinumo Creek	AZ	12	10	2									
	Conf. Little Colorado	AZ	18	3	15									
Virgin River	Beaver Dam Wash	UT	10	10										
	Meadow Valley Wash	NV	1							1				
Other Sites			66	5	11	3	14			8	15	6	4	
Total			409	76	120	13	42	5	4	9	15	6	4	115

## Appendix B

### The grouping of regional sites with hybridization

Six of the nine regions of potential hybridization are located within the Green River Basin, the largest tributary to the Upper Colorado River. Three of these are above Flaming Gorge Dam (located at the Utah/Wyoming border) and contain native Flannelmouth Sucker (*Catostomus latipinnis*) and introduced White Sucker (*C. commersonii*). The Big Sandy River provides habitat for native Bluehead Sucker (*C. Pantosteus discobolus*) and Mountain Sucker (*C. P. platyrhynchus*), as well as introduced Longnose Sucker (*C. catostomus*). Blacks Fork is represented by native Bluehead Sucker, Mountain Sucker, and their potential hybrids. The remaining sites above Flaming Gorge Dam (Upper Green River region) drain into the mainstem Green River and contain very few native Bluehead Sucker and Mountain Sucker, a residual of ill‐fated attempts in 1962 to remove native fish for the purposes of establishing a Rainbow Trout fishery (Hilton & Smith, 2014; Holden, 1991).

The remaining sites in the Green River Basin include (a) Yampa River/Little Snake River, a source of previous hybrid studies between White Sucker, Flannelmouth Sucker, and Bluehead Sucker (Douglas & Douglas, 2010; McDonald et al., 2008); (b) Middle Green River, which does not contain White Sucker but potential hybrids between Flannelmouth Sucker and Bluehead Sucker; and (c) Price River, with a population of Mountain Sucker (Sigler & Miller, 1963) introduced from the Bonneville Basin that may also be hybridizing with native Bluehead Sucker.

Samples from the San Juan River were collected from the Navajo River, Arch Canyon, and the mainstem San Juan River. White Sucker is rare in the San Juan River, with exception of perennial tributaries such as the Animas and Navajo rivers (Carman, 2007). In a range‐wide analysis of Bluehead Sucker, researchers found haplotypes of Desert Sucker (*C. P. clarkii*) throughout Upper Colorado River Basin, but especially so in Arch Canyon, an isolated population within the San Juan River drainage (Hopken et al., 2013). The mainstem San Juan River supports native Flannelmouth and Bluehead suckers, and is a recovery site for the endangered Razorback Sucker (*Xyrauchen texanus*), a species that will hybridize with Flannelmouth Sucker (Douglas & Marsh, 1998; Carman, 2007).

The Grand Canyon and Virgin River link the Lower and the Upper Colorado River basins, and as such may represent the potential mixing zone of sister species between the basins. This would include Sonora Sucker (*C. insignis*) × Flannelmouth Sucker, and Desert Sucker × Bluehead Sucker. Hybridization between Razorback Sucker and Flannelmouth Sucker has also been detected in this region (Douglas & Marsh, 1998).

The Little Colorado River was separated from the Colorado River Basin for 20kya by Grand Falls (Duffield et al., 2006) and was thus not included in this study. However, hybridization has also occurred in the Little Colorado River (Bangs, Douglas, Chafin, & Douglas, 2020).

## References

[eva13042-bib-0001] Abbott, R. J. , Barton, N. H. , & Good, J. M. (2016). Genomics of hybridization and its evolutionary consequences. Molecular Ecology, 25, 2325–2332. 10.1111/mec.13685 27145128

[eva13042-bib-0002] Allendorf, F. W. , Hohenlohe, P. A. , & Luikart, G. (2010). Genomics and the future of conservation genetics. Nature Reviews Genetics, 11, 697 10.1038/nrg2844 20847747

[eva13042-bib-0003] Allendorf, F. W. , Leary, R. F. , Spruell, P. , & Wenburg, J. K. (2001). The problems with hybrids: Setting conservation guidelines. Trends in Ecology and Evolution, 16, 613–622. 10.1016/S0169-5347(01)02290-X

[eva13042-bib-0004] Anderson, E. C. , & Thompson, E. A. (2002). A model‐based method for identifying species hybrids using multilocus genetic data. Genetics, 160, 1217–1229. 11901135 10.1093/genetics/160.3.1217 PMC1462008

[eva13042-bib-0005] Arnold, M. L. (1992). Natural hybridization as an evolutionary process. Annual Review of Ecology and Systematics, 23, 237–261. 10.1146/annurev.es.23.110192.001321

[eva13042-bib-0006] Arnold, M. L. (2016). Divergence with genetic exchange. Oxford, UK: Oxford University Press.

[eva13042-bib-0007] Bangs, M. R. , Douglas, M. R. , Chafin, T. K. , & Douglas, M. E. (2020). Gene flow and species delimitation in fishes of Western North America: Flannelmouth (*Catostomus latipinnis*) and Bluehead sucker (*C. Pantosteus discobolus*). Ecology and Evolution. 10.1002/ece3.6384. [Epub ahead of print]. PMC7381754 32724527

[eva13042-bib-0008] Bangs, M. R. , Douglas, M. R. , Mussmann, S. M. , & Douglas, M. E. (2018). Unraveling historical introgression and resolving phylogenetic discord within *Catostomus* (Osteichthys: Catostomidae). BMC Evolutionary Biology, 18, 86 10.1186/s12862-018-1197-y 29879898 PMC5992631

[eva13042-bib-0009] Bangs, M. R. , Douglas, M. R. , Thompson, P. , & Douglas, M. E. (2017). Anthropogenic impacts facilitate native fish hybridization in the Bonneville Basin of western North America. Transactions of the American Fisheries Society, 146, 16–21. 10.1080/00028487.2016.1235611

[eva13042-bib-0011] Benestan, L. M. , Ferchaud, A. L. , Hohenlohe, P. A. , Garner, B. A. , Naylor, G. J. , Baums, I. B. , … Luikart, G. (2016). Conservation genomics of natural and managed populations: Building a conceptual and practical framework. Molecular Ecology, 25, 2967–2977. 10.1111/mec.13647 27086132

[eva13042-bib-0012] Bhattacharya, T. , Tierney, J. E. , Addison, J. A. , & Murray, J. W. (2018). Ice‐sheet modulation of deglacial North American monsoon intensification. Nature Geoscience, 11, 848–852. 10.1038/s41561-018-0220-7

[eva13042-bib-0013] Bosley, C. E. (1960). Pre‐impoundment study of the Flaming Gorge Reservoir. Fisheries Technical Report 9, Wyoming Game and Fish Department, Cheyenne. Retrieved from http://www.nativefishlab.net/library/textpdf/17734.pdf.

[eva13042-bib-0014] Brunner, P. C. , Douglas, M. R. , & Bernatchez, L. (1998). Microsatellite and mitochondrial DNA assessment of population structure and stocking effects in Arctic charr *Salvelinus alpinus* (Teleostei: Salmonidae) from central Alpine lakes. Molecular Ecology, 7, 209–223. 10.1046/j.1365-294x.1998.00341.x

[eva13042-bib-0015] Brunner, P. C. , Douglas, M. R. , Osinov, A. , Wilson, C. C. , & Bernatchez, L., (2001). Holarctic phylogeography of Arctic char (*Salvelinus alpinus* L.) inferred from mitochondrial DNA sequences. Evolution, 55, 573–586. 10.1111/j.0014-3820.2001.tb00790.x 11327164

[eva13042-bib-0016] Bryson, R. W. , Murphy, R. W. , Lathrop, A. , & Lazcano‐Villareal, D. (2011). Evolutionary drivers of phylogeographical diversity in the highlands of Mexico: A case study of the *Crotalus triseriatus* species group of montane rattlesnakes. Journal of Biogeography, 38, 697–710. 10.1111/j.1365-2699.2010.02431

[eva13042-bib-0017] Buth, D. G. , Haglund, T. R. , & Minckley, W. L. (1992). Duplicate gene expression and allozyme divergence diagnostic for *Catostomus tahoensis* and endangered *Chasmistes cujus* in Pyramid Lake, Nevada. Copeia, 1994, 935–941.

[eva13042-bib-0018] Buth, D. G. , Murphy, R. W. , & Ulmer, L. (1987). Population differentiation and introgressive hybridization of the flannelmouth sucker and of hatchery and native stocks of the razorback sucker. Transactions of the American Fisheries Society, 116, 103–110. 10.1577/1548-8659(1987)116<103:PDAIHO>2.0.CO;2

[eva13042-bib-0019] Cayan, D. R. , Das, T. , Pierce, D. W. , Barnett, T. P. , Tyree, M. , & Gershunov, A. (2010). Future dryness in the southwest US and the hydrology of the early 21st century drought. Proceedings of the National Academy of Sciences of the United States of America, 107, 21271–21276. 10.1073/pnas.0912391107. 21149687 PMC3003012

[eva13042-bib-0020] Chafin, T. K. , Douglas, M. R. , Bangs, M. R. , Mussmann, S. M. & Douglas, M. E. . (2019). Taxonomic uncertainty and phylogenomics: Rescuing a contentious species complex from the anomaly zone. bioRxiv. http://biorxiv.org/cgi/content/short/692509v1 10.1093/gbe/evab200 PMC8449829 34432005

[eva13042-bib-0021] Chafin, T. K. , Douglas, M. R. , Martin, B. T. , & Douglas, M. E. (2019). Hybridization drives genetic erosion in sympatric desert fishes of western North America. Heredity, 123(6), 759–773. 10.1038/s41437-019-0259-2. 31431737 PMC6834602

[eva13042-bib-0022] Chen, W. J. , & Mayden, R. L. (2012). Phylogeny of suckers (Teleostei: Cypriniformes: Catostomidae): Further evidence of relationships provided by the single‐copy nuclear gene IRBP2. Zootaxa, 3586, 195–210.

[eva13042-bib-0023] Clarkson, R. W. , & Minckley, W. L. (1988). Morphology and foods of Arizona catostomid fishes: *Catostomus insignis*, *Pantosteus clarkii*, and their putative hybrids. Copeia, 1988, 422–433.

[eva13042-bib-0024] Coyne, J. A. , & Orr, H. A. (2004). Speciation. Sunderland, MA: Sinauer Associates.

[eva13042-bib-0025] Darras, H. , Leniaud, L. , & Aron, S. (2014). Large‐scale distribution of hybridogenetic lineages in a Spanish desert ant. Proceedings of the Royal Society B: Biological Sciences, 281, 20132396 10.1098/rspb.2013.2396. PMC3843834 24225458

[eva13042-bib-0026] Dasmahapatra, K. K. , Walters, J. R. , Briscoe, A. D. , Davey, J. W. , Whibley, A. , Nadeau, N. J. , … & Jiggins, C. D. (2012). Butterfly genome reveals promiscuous exchange of mimicry adaptations among species. Nature, 487, 94–98. 10.1038/nature11041 22722851 PMC3398145

[eva13042-bib-0027] Devitt, T. J. , Wright, A. M. , Cannatella, D. C. , & Hillis, D. M. (2019). Species delimitation in endangered groundwater salamanders: Implications for aquifer management and biodiversity conservation. Proceedings of the National Academy of Sciences of the United States of America, 116, 2624–2633. 10.1073/pnas.1815014116. 30642970 PMC6377464

[eva13042-bib-0028] Douglas, M. R. , & Brunner, P. C. (2002). Biodiversity of Central Alpine *Coregonus* (Salmoniformes): Impact of one‐hundred years of management. Ecological Applications, 12, 154–172. 10.1890/1051-0761(2002)012[0154:BOCACS]2.0.CO;2

[eva13042-bib-0029] Douglas, M. R. , Brunner, P. C. , & Bernachez, L. (1999). Do assemblages of *Coregonus* (Teleostei: Salmoniformes) in the Central Alpine region of Europe represent species flocks? Molecular Ecology, 8, 589–603. 10.1046/j.1365-294x.1999.00581.x

[eva13042-bib-0030] Douglas, M. R. , Brunner, P. C. , & Douglas, M. E. (2003). Drought in an evolutionary context: Molecular variability in Flannelmouth Sucker (*Catostomus latipinnis*) from the Colorado River Basin of western North American. Freshwater Biology, 48, 1254–1273. 10.1046/j.1365-2427.2003.01088.x

[eva13042-bib-0031] Douglas, M. R. , Davis, M. A. , Amarello, M. , Smith, J. J. , Schuett, G. W. , Herrmann, H.‐W. , … Douglas, M. E. (2016). Anthropogenic impacts drive niche and conservation metrics of a cryptic rattlesnake on the Colorado Plateau of western North America. Royal Society Open Science, 3, 160047 10.1098/rsos.160047 27152218 PMC4852641

[eva13042-bib-0032] Douglas, M. R. , & Douglas, M. E. (2010). Molecular approaches to stream fish ecology. American Fisheries Society Symposium, 73, 157–195.

[eva13042-bib-0033] Douglas, M. E. , Douglas, M. R. , Schuett, G. W. , & Porras, L. W. (2006). Evolution of rattlesnakes (Viperidae; Crotalus) in the warm deserts of western North America shaped by Neogene vicariance and Quaternary climate change. Molecular Ecology, 15, 3353–3374. 10.1111/j.1365-294X.2006.03007.x 16968275

[eva13042-bib-0034] Douglas, M. E. , Douglas, M. R. , Schuett, G. W. , & Porras, L. W. (2009). Climate change and evolution of the New World pitviper genus *Agkistrodon* (Viperidae). Journal of Biogeography, 36, 1164–1180. 10.1111/j.1365-2699.2008.02075.x

[eva13042-bib-0035] Douglas, M. E. , & Marsh, P. C. (1998). Population and survival estimates of *Catostomus latipinnis* in northern Grand Canyon, with distribution and abundance of hybrids with *Xyrauchen texanus* . Copeia, 1998, 915–925. 10.2307/1447338

[eva13042-bib-0036] Dowling, T. E. , Markle, D. F. , Tranah, G. J. , Carson, E. W. , Wagman, D. W. , & May, B. P. (2016). Introgressive hybridization and the evolution of lake‐adapted catostomid fishes. PLoS One, 11, e0149884 10.1371/journal.pone.0149884 26959681 PMC4784955

[eva13042-bib-0037] Dowling, T. E. , Minckley, W. L. , & Marsh, P. C. (1996). Mitochondrial DNA diversity within and among populations of razorback sucker (*Xyrauchen texanus*) as determined by restriction endonuclease analysis. Copeia, 1996, 542–550. 10.2307/1447518

[eva13042-bib-0038] Dowling, T. E. , & Secor, C. L. (1997). The role of hybridization and introgression in the diversification of animals. Annual Review of Ecology and Systematics, 28, 593–619. 10.1146/annurev.ecolsys.28.1.593

[eva13042-bib-0039] Dowling, T. E. , Turner, T. F. , Carson, E. W. , Saltzgiver, M. J. , Adams, D. , Kesner, B. , & Marsh, P. C. (2014). Time‐series analysis reveals genetic responses to intensive management of razorback sucker (*Xyrauchen texanus*). Evolutionary Applications, 7, 339–354. 10.1111/eva.12125 24665337 PMC3962295

[eva13042-bib-0040] Du Rietz, G. E. (1930). The fundamental units of biological taxonomy. Svensk Botanisk Tidskrift, 24, 333–428.

[eva13042-bib-0041] Eaton, D. A. , & Ree, R. H. (2013). Inferring phylogeny and introgression using RADseq data: An example from flowering plants (*Pedicularis*: Orobanchaceae). Systematic Biology, 62, 689–706. 10.1093/sysbio/syt032 23652346 PMC3739883

[eva13042-bib-0042] Edelman, N. B. , Frandsen, P. B. , Miyagi, M. , Clavijo, B. , Davey, J. , & Dikow, R. B. … Mallet, J. (2019). Genomic architecture and introgression shape a butterfly radiation. Science, 366, 594–599. 10.1126/science.aaw2090 31672890 PMC7197882

[eva13042-bib-0043] Edmands, S. (2002). Does parental divergence predict reproductive compatibility? Trends in Ecology and Evolution, 17, 520–527. 10.1016/S0169-5347(02)02585-5

[eva13042-bib-0044] Ericson, P. G. P. , Qu, Y. , Rasmussen, P. C. , Blom, M. P. K. , Rheindt, F. E. , & Irestedt, M. (2019). Genomic differentiation tracks earth historic isolation in an Indo‐Australasian archipelagic pitta (Pittidae; Aves) complex. BMC Evolutionary Biology, 19, 151 10.1186/s12862-019-1481-5 31340765 PMC6657069

[eva13042-bib-0045] Evanno, G. , Regnaut, S. , & Goudet, J. (2005). Detecting the number of clusters of individuals using the software STRUCTURE: A simulation study. Molecular Ecology, 14, 2611–2620. 10.1111/j.1365-294X.2005.02553.x 15969739

[eva13042-bib-0047] Felsenstein, J. (1993) Phylip (Phylogeny Inference Package), version 3.5c. Retrieved from http://evolution.genetics.washington.edu/phylip.html.

[eva13042-bib-0048] Ficke, A. D. , Myrick, C. A. , & Hansen, L. J. (2007). Potential impacts of global climate change on freshwater fisheries. Reviews in Fish Biology and Fisheries, 17, 581–613. 10.1007/s11160-007-9059-5

[eva13042-bib-0049] Fisher, R. A. (1930). The genetical theory of natural selection. Oxford, UK: Clarendon.

[eva13042-bib-0050] Fontaine, M. C. , Pease, J. B. , Steele, A. , Waterhouse, R. M. , Neafsey, D. E. , Sharakhov, I. V. , … Besansky, N. J. (2015). Extensive introgression in a malaria vector species complex revealed by phylogenomics. Science, 347, 1258524 10.1126/science.1258524 25431491 PMC4380269

[eva13042-bib-0051] Funk, D. J. , Nosil, P. , & Etges, W. J. (2006). Ecological divergence exhibits consistently positive associations with reproductive isolation across disparate taxa. Proceedings of the National Academy of Sciences, 103(9), 3209–3213. 10.1073/pnas.0508653103. PMC1413886 16492742

[eva13042-bib-0052] Funk, W. C. , McKay, J. K. , Hohenlohe, P. A. , & Allendorf, F. W. (2012). Harnessing genomics for delineating conservation units. Trends in Ecology & Evolution, 27(9), 489–496. 10.1016/j.tree.2012.05.012 22727017 PMC4185076

[eva13042-bib-0053] Garner, B. A. , Hand, B. K. , Amish, S. J. , Bernatchez, L. , Foster, J. T. , Miller, K. M. , … Luikart, G. (2016). Genomics in conservation: Case studies and bridging the gap between data and application. Trends in Ecology & Evolution, 31, 81–83. 10.1016/j.tree.2015.10.009 26654124

[eva13042-bib-0054] Gompert, Z. , & Buerkle, C. A. (2009). A powerful regression‐based method for admixture mapping of isolation across the genome of hybrids. Molecular Ecology, 18, 1207–1224. 10.1111/j.1365-294X.2009.04098.x 19243513

[eva13042-bib-0055] Gompert, Z. , & Buerkle, C. A. (2010). introgress: A software package for mapping components of isolation in hybrids. Molecular Ecology Resources, 10, 378–384. 10.1111/j.1755-0998.2009.02733.x 21565033

[eva13042-bib-0056] Good, J. M. , Demboski, J. R. , Nagorsen, D. W. , & Sullivan, J. (2003). Phylogeography and introgressive hybridization: Chipmunks (genus *Tamias*) in the northern Rocky Mountains. Evolution, 57, 1900–1916. 10.1111/j.0014-3820.2003.tb00597.x 14503631

[eva13042-bib-0057] Haig, S. M. , & Allendorf, F. W. (2006). Hybrids and policy In Scott J. M., Goble D. D., & Davis F. W. (Eds.), Endangered Species Act at Thirty, Volume 2: Conserving biodiversity in human‐dominated landscapes (pp. 150–163). Washington, DC: Island Press.

[eva13042-bib-0058] Hamilton, J. A. , & Miller, J. M. (2016). Adaptive introgression as a resource for management and genetic conservation in a changing climate. Conservation Biology, 30, 33–41. 10.1111/cobi.12574 26096581

[eva13042-bib-0059] Harrison, R. G. (2012). The language of speciation. Evolution, 66, 3643–3657. 10.1111/j.1558-5646.2012.01785.x 23206125

[eva13042-bib-0061] Hinck, J. E. , Blazer, V. S. , Denslow, N. D. , Echols, K. R. , Gross, T. S. , May, T. W. , … Tillitt, D. E. (2007). Chemical contaminants, health indicators, and reproductive biomarker responses in fish from the Colorado River and its tributaries. Science of the Total Environment, 378, 376–402. 10.1016/j.scitotenv.2007.02.032 17418376

[eva13042-bib-0062] Holden, P. H. (1991). Ghosts of the Green River: Impacts of Green River poisoning on management of native fishes In Minckley W. L., & Deacon J. E. (Eds.), Battle against extinction: Native fish management in the American West (pp. 43–54). Tucson: University of Arizona Press https://repository.arizona.edu/handle/10150/625250.

[eva13042-bib-0063] Holden, P. B. , & Stalnaker, C. B. (1975). Distribution and abundance of mainstream fishes of the middle and upper Colorado River basins, 1967–1973. Transactions of the American Fisheries Society, 104, 217–231. 10.1577/1548-8659(1975)104<217:DAAOMF>2.0.CO;2

[eva13042-bib-0064] Hopken, M. W. , Douglas, M. R. , & Douglas, M. E. (2013). Stream hierarchy defines riverscape genetics of a North American desert fish. Molecular Ecology, 956–971, 10.1111/mec.12156 23279045

[eva13042-bib-0065] Hubbs, C. L. , Hubbs, L. C. , & Johnson, R. E. (1943). Hybridization in Nature between Species of Catostomid Fishes. Ann Arbor, MI: University of Michigan Press Retrieved from https://babel.hathitrust.org/cgi/pt?id=mdp.39015089812468&view=thumb&seq=1.

[eva13042-bib-0066] Kane, N. C. , King, M. G. , Baker, M. S. , Raduski, A. , Karrenberg, S. , Yatabe, Y. , … Rieseberg, L. H. (2009). Comparative genomic and population genetic analyses indicate highly porous genomes and high levels of gene flow between divergent *Helianthus* species. Evolution, 63, 2061–2075. 10.1111/j.1558-5646.2009.00703.x 19473382 PMC2731706

[eva13042-bib-0067] Kohli, B. A. , Fedorov, V. B. , Waltari, E. , & Cook, J. A. (2015). Phylogeography of a Holarctic rodent (*Myodes rutilus*): Testing high‐latitude biogeographical hypotheses and the dynamics of range shifts. Journal of Biogeography, 42, 377–389. 10.1111/jbi.12433

[eva13042-bib-0068] Licona‐Vera, Y. , Ornelas, J. F. , Wethington, S. , & Bryan, K. B. (2018). Pleistocene range expansions promote divergence with gene flow between migratory and sedentary populations of *Calothorax* hummingbirds. Biological Journal of the Linnean Society, 124, 645–667. 10.1093/biolinnean/bly084

[eva13042-bib-0069] Lowe, W. H. , Muhlfeld, C. C. , & Allendorf, F. W. (2015). Spatial sorting promotes the spread of maladaptive hybridization. Trends in Ecology & Evolution, 30, 456–462. 10.1016/j.tree.2015.05.008 26122483

[eva13042-bib-0070] Mandeville, E. G. , Parchman, T. L. , McDonald, D. B. , & Buerkle, C. A. (2015). Highly variable reproductive isolation among pairs of *Catostomus* species. Molecular Ecology, 24, 1856–1872. 10.1111/mec.13118 25703195 PMC4390503

[eva13042-bib-0071] Mandeville, E. G. , Parchman, T. L. , Thompson, K. G. , Compton, R. I. , Gelwicks, K. R. , Song, S. J. , & Buerkle, C. A. (2017). Inconsistent reproductive isolation revealed by interactions between Catostomus fish species. Evolution Letters, 1, 255–268. 10.1002/evl3.29 30283654 PMC6121845

[eva13042-bib-0072] McAda, C. W. , & Wydoski, R. S. (1980). The razorback sucker, Xyrauchen texanus, in the upper Colorado River basin, 1974–76. Technical Paper 99. U.S. Fish and Wildlife Service. Retrieved from http://pubs.er.usgs.gov/publication/tp99.

[eva13042-bib-0073] McDonald, D. B. , Parchman, T. L. , Bower, M. R. , Hubert, W. A. , & Rahel, F. J. (2008). An introduced and a native vertebrate hybridize to form a genetic bridge to second native species. Proceedings of the National Academy of Sciences of the United States of America, 105, 10837–10842. 10.1073/pnas.0712002105. 18658235 PMC2504823

[eva13042-bib-0074] Michel, A. P. , Sim, S. , Powell, T. H. Q. , Taylor, M. S. , Nosil, P. , & Feder, J. L. (2010). Widespread genomic divergence during sympatric speciation. Proceedings of the National Academy of Sciences of the United States of America, 107, 9724–9729. 10.1073/pnas.1000939107. 20457907 PMC2906890

[eva13042-bib-0075] Migliore, J. , Kaymak, E. , Mariac, C. , Couvreur, T. L. P. , Lissambo, B.‐J. , Piñeiro, R. , & Hardy, O. J. (2019). Pre‐Pleistocene origin of phylogeographical breaks in African rain forest trees: New insights from *Greenwayodendron* (Annonaceae) phylogenomics. Journal of Biogeography, 46, 212–223. 10.1111/jbi.13476

[eva13042-bib-0076] Minckley, W. L. (1980). Morphological variation in catostomid fishes of the Grand Canyon Region, middle Colorado River basin. Final Report, U.S. National Park Service Contract, Grand Canyon National Park, Grand Canyon, Arizona. Tempe: Arizona State University Retrieved from http://www.nativefishlab.net/library/textpdf/13689.pdf.

[eva13042-bib-0077] Minckley, W. L. (1983). Status of the razorback sucker, *Xyrauchen texanus* (Abbott), in the lower Colorado River basin. The Southwestern Naturalist, 1, 165–187. 10.2307/3671385

[eva13042-bib-0078] Minckley, W. L. (1995). Translocation as a tool for conserving imperiled fishes: Experiences in western United States. Biological Conservation, 72, 297–309. Retrieved from https://www.fs.fed.us/rm/boise/publications/BTWorkshop/Minckley%201995.pdf

[eva13042-bib-0079] Minckley, W. L. , Hendrickson, D. A. , & Bond, C. E. (1986). Geography of western North American freshwater fishes; Description and relationships to intracontinental tectonism In Hocutt C. H., & Wiley E. O. (Eds.), The Zoogeography of North American Freshwater Fishes (pp. 519–613). New York, NY: John Wiley & Sons.

[eva13042-bib-0080] Morales‐Barbero, J. , Martinez, P. A. , Ferrer‐Castán, D. , & Olalla‐Tárraga, M. Á. (2018). Quaternary refugia are associated with higher speciation rates in mammalian faunas of the western Palaearctic. Ecography, 41, 607–621. 10.1111/ecog.02647

[eva13042-bib-0081] Nadeau, N. J. , Whibley, A. , Jones, R. T. , Davey, J. W. , Dasmahapatra, K. K. , Baxter, S. W. , … Jiggins, C. D. (2012). Genomic islands of divergence in hybridizing *Heliconius* butterflies identified by large‐scale targeted sequencing. Philosophical Transactions of the Royal Society London B Biological Sciences, 367, 343–353. 10.1098/rstb.2011.0198 PMC3233711 22201164

[eva13042-bib-0082] Nelson, G. J. (1968). Hybridization and isolating mechanisms between *Catostomus commersonii* and *C. macrocheilus* (Pisces: Catostomidae). Journal of the Fisheries Research Board of Canada, 25, 101–150. 10.1139/f68-008

[eva13042-bib-1003] Nosil, P. , Funk, D. J. , & Ortiz‐Barrientos, D. A. N. I. E. L. (2009). Divergent selection and heterogeneous genomic divergence. Molecular Ecology, 18(3), 375–402. 19143936 10.1111/j.1365-294X.2008.03946.x

[eva13042-bib-0083] Pritchard, J. K. , Stephens, M. , & Donnelly, P. (2000). Inference of population structure using multilocus genotype data. Genetics, 155, 945–959. PMC1461096. 10835412 10.1093/genetics/155.2.945 PMC1461096

[eva13042-bib-0084] Quartarone, F. (1995). Historical accounts of upper Colorado River basin endangered fish. Final Report. Revised Edition, U.S. Fish and Wildlife Service, Denver Federal Center, Denver, Colorado. Retrieved from https://babel.hathitrust.org/cgi/pt?id=uc1.31210024952853&view=1up&seq=1

[eva13042-bib-0085] Quist, M. C. , Bower, M. R. , Hubert, W. A. , Parchman, T. L. , & McDonald, D. B. (2009). Morphometric and meristic differences among bluehead suckers, flannelmouth suckers, white suckers, and their hybrids: Tools for the management of native species in the upper Colorado River basin. North American Journal of Fisheries Management, 29, 460–467. 10.1577/M08-098.1

[eva13042-bib-0086] Rhymer, J. , & Simberloff, D. (1996). Extinction by hybridization and introgression. Annual Review of Ecology and Systematics, 27, 83–109. 10.1146/annurev.ecolsys.27.1.83

[eva13042-bib-0087] Seehausen, O. , Butlin, R. K. , Keller, I. , Wagner, C. E. , Boughman, J. W. , Hohenlohe, P. A. , … Widmer, A. (2014). Genomics and the origin of species. Nature Review Genetics, 15, 176–192. 10.1038/nrg3644 24535286

[eva13042-bib-0088] Sigler, W. F. , & Miller, R. R. (1963). Fishes of Utah. Salt Lake City, UT: Utah State Department of Fish and Game.

[eva13042-bib-0089] Smith, G. R. (1966). Distribution and evolution of the North American catostomid fishes of the subgenus *Pantosteus*, genus *Catostomus* . Miscellaneous Publications Museum of Zoology University of Michigan, 129, 1–132.

[eva13042-bib-0090] Smith, G. R. , Hall, J. G. , Koehn, R. K. , & Innes, D. J. (1983). Taxonomic relationships of the Zuni Mountain Sucker, *Catostomus discobolus yarrowi* . Copeia, 1983, 37–48. 10.2307/1444696

[eva13042-bib-0091] Smith, G. R. , Stewart, J. D. , & Carpenter, N. E. (2013). Fossil and recent mountain suckers *Pantosteus*, and significance of introgression in catostomid fishes of Western United States. Occasional Papers Museum of Zoology University of Michigan, 724, 1–59.

[eva13042-bib-0092] Smith, G. R. , Zaroban, D. W. , High, B. , Sigler, J. W. , Schilling, J. , Krabbenhoft, T. J. , & Dowling, T. E. (2018) Introgressive mtDNA transfer in hybrid lake suckers (Teleostei, Catostomidae) in Western United States. Miscellaneous Publications Museum of Zoology University of Michigan, 204, 87–117.

[eva13042-bib-0093] Spencer, J. E. , Smith, G. R. , & Dowling, T. E. (2008). Middle to late Cenozoic geology, hydrography, and fish evolution in the American Southwest In Reheis M. C., Hershler R., & Miller D. M. (Eds.), Late Cenozoic Drainage history of the Southwestern Great Basin and Lower Colorado River Region: Geologic and biotic perspectives (pp. 279–299). The Geological Society of America, Inc. Geological Society of America Special Paper 439.

[eva13042-bib-0094] Sublette, J. E. , Hatch, M. D. , & Sublette, M. S. (1990). The fishes of New Mexico. Albuquerque, NM: University of New Mexico Press Retrieved from http://www.nativefishlab.net/library/textpdf/14664.pdf.

[eva13042-bib-1000] Sullivan, R. , van Hengstum, P. J. , Winkler, T. S. , Donnelly, J. P. , Albury, N. A. , & Steadman, D. W. (2016). A sedimentary record of middle Holocene precipitation and terrestrial vertebrates from Great Cistern Blue Hole (Abaco Island), The Bahamas. AGUFM, 2016, PP33B–2365.

[eva13042-bib-0095] Tranah, G. J. , & May, B. (2006). Patterns of intra‐ and interspecies genetic diversity in Klamath River Basin Suckers. Transactions of the American Fisheries Society, 135, 306–316. 10.1577/T05-026.1

[eva13042-bib-0096] Unmack, P. J. , Dowling, T. E. , Laitinen, N. J. , Secor, C. L. , Mayden, R. L. , Shiozawa, D. K. , & Smith, G. R. (2014). Influence of introgression and geological processes on phylogenetic relationships of western North American mountain suckers (*Pantosteus*, Catostomidae). PLoS One, 9, e90061 10.1371/journal.pone.0090061 24619087 PMC3949674

[eva13042-bib-0046] US Fish and Wildlife Service . (1991). Endangered and threatened wildlife and plants; the Razorback Sucker (*Xyrauchen texanus*) determined to be an endangered species. Federal Register, 56(205), 54957–54967.

[eva13042-bib-0097] Uyeno, T. , & Miller, R. R. (1965). Middle Pliocene cyprinid fishes from the Bidahochi Formation, Arizona. Copeia, 1965, 28–41. 10.2307/1441236

[eva13042-bib-0098] vonHoldt, B. M. , Brzeski, K. E. , Wilcove, D. S. , & Rutledge, L. Y. (2018). Redefining the role of admixture and genomics in species conservation. Conservation Letters, 11, e12371 10.1111/conl.12371

[eva13042-bib-0099] Wendel, J. F. , & Doyle, J. J. (1998). Phylogenetic incongruence: Window into genome history and molecular evolution In Soltis D. E., Soltis P. S., & Doyle J. J. (Eds.), Molecular systematics of plants II (pp. 265–296). Boston MA: Springer Publishers.

[eva13042-bib-0100] Wiley, R. W. (2008). The 1962 rotenone treatment of the Green River, Wyoming and Utah, revisited: Lessons learned. Fisheries, 33, 611–617. 10.1577/1548-8446-33.12.611

