# Supplementary material for: Reticulate evolution as a management challenge: Patterns of admixture with phylogenetic distance in endemic fishes of western North America
Source: Evol Appl. 2020 Jun 29;13(6):1400–19. doi: 10.1111/eva.13042 (PMC7359839; doi:10.1111/eva.13042)
Supplement: Supplementary file 1 — Figures S1‐S2 [file EVA-13-1400-s001.pdf]

A

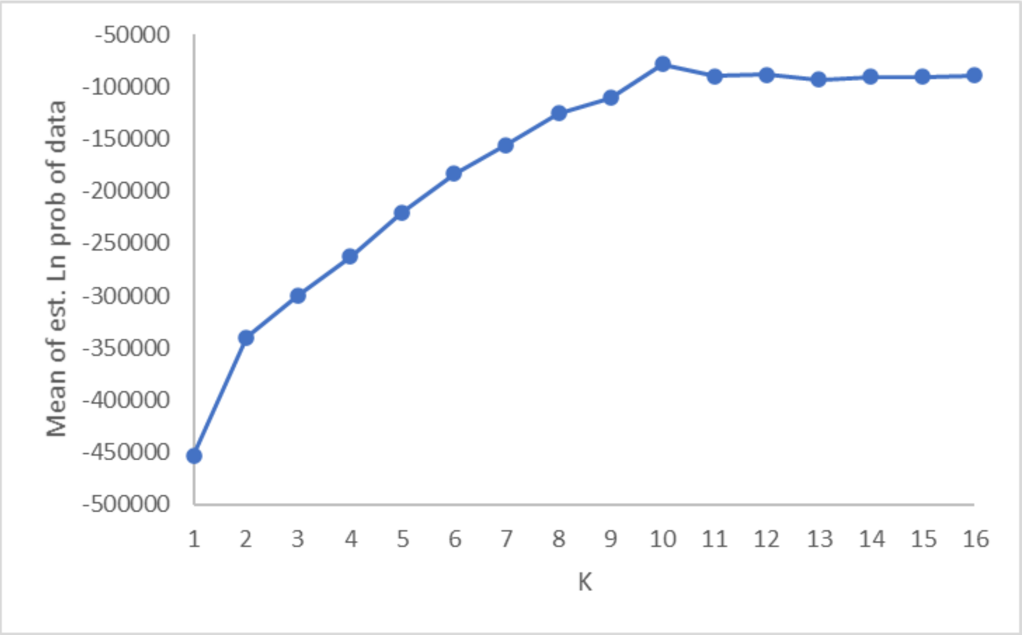

B

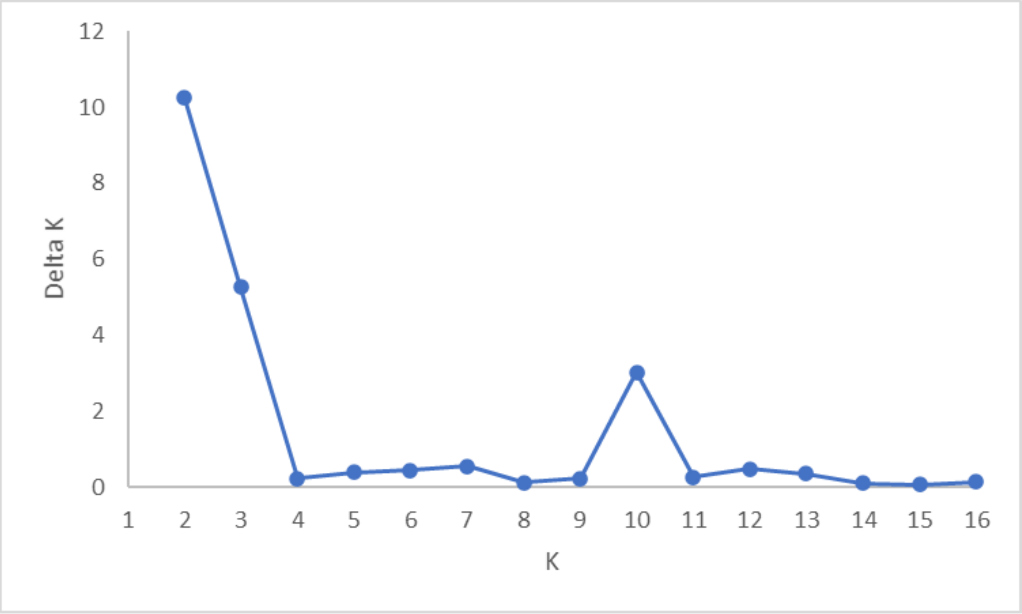

Supplemental Figure 1: (A) Mean natural log probabilities of data across 15 replicates against number of clusters (K) for the Structure runs. (B) Delta K (as calculated per Evanno et al. 2005) across 15 replicates against number of clusters (K) for the Structure runs.

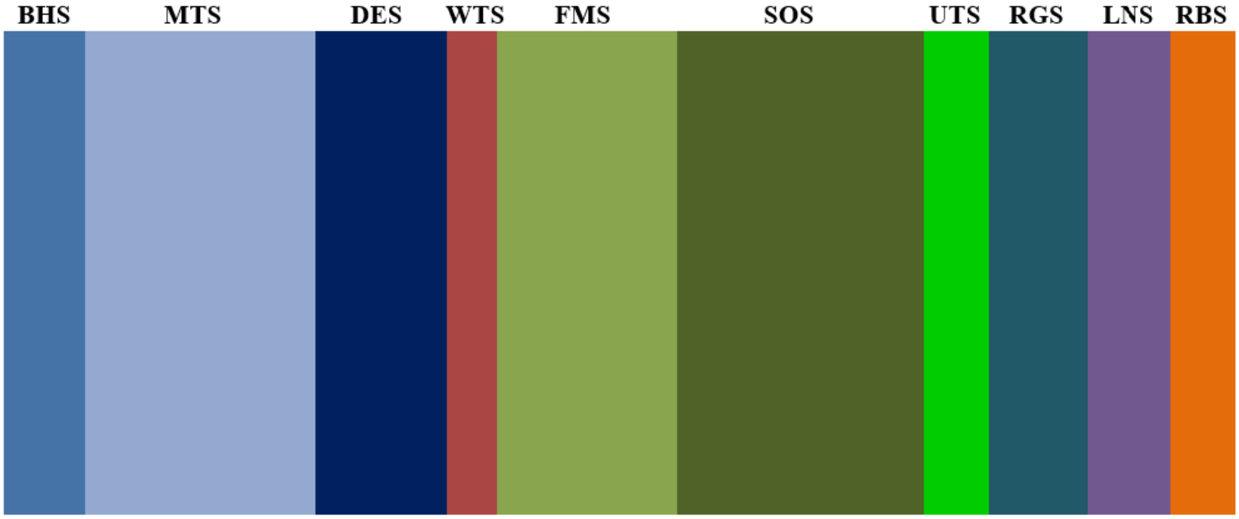

Supplemental Figure 2: Structure plot for the 66 reference samples caught outside of the hybrid zones along with five Longnose Sucker (LNS) from the Big Sandy and four Razorback Sucker (RBS) from the San Juan River. These samples were all used in the phylogenomic study of *Catostomus* (Bangs et al. 2018) where sampling locations are detailed. All reference samples assigned to only one cluster and are colored in the same matter as Figure 2. Abbreviations include Bluehead Sucker (BHS), Mountain Sucker (MTS), Desert Sucker (DES), White Sucker (WTS), Flannelmouth Sucker (FMS), Sonora Sucker (SOS), Utah Sucker (UTS), Rio Grande Sucker (RGS), Longnose Sucker (LNS), and Razorback Sucker (RBS).
